# Integrative Single-Cell and Bulk Transcriptomic Analysis Reveals Ribosome Biogenesis-Associated Molecular Subtypes and Immune Characteristics in Hepatocellular Carcinoma

**DOI:** 10.3390/biology15212034

**Published:** 2026-11-08

**Authors:** Zejing Liu, Shangdi Zhang, Hongwei Gao, Yicheng Ma, Shan Gao, Jing Yang, Linjing Li

**Affiliations:** Pharmacogenomics Laboratory, Laboratory Medicine Center, The Second Hospital & Clinical Medical School, Lanzhou University, Lanzhou City 730030, China; lzejing2025@lzu.edu.cn (Z.L.);

**Keywords:** hepatocellular carcinoma, ribosome biogenesis, tumor immune microenvironment, molecular subtypes, single-cell transcriptomics

## Abstract

Hepatocellular carcinoma (HCC) exhibits substantial molecular heterogeneity. We integrated single-cell and bulk transcriptomic data to investigate associations between ribosome biogenesis-related (RiboSis-related) gene expression and the molecular and immune characteristics of HCC. GLUL^+^ hepatocytes had the highest RiboSis-related gene-set score among hepatocyte subpopulations, and ligand–receptor analysis inferred an MDK–SDC2 interaction between ALDOA^+^ and GLUL^+^ hepatocytes. Feature selection identified four candidate genes, with subsequent subtype analysis highlighting BOP1 and EXOSC4. The two molecular subtypes differed in RiboSis-related gene expression, immune cell signature scores, mutation profiles, checkpoint gene expression, and TIDE scores. Immunohistochemistry showed higher BOP1 and EXOSC4 staining in HCC than in paired adjacent tissues. EXOSC4 staining was lower in HBV-associated than in non-viral HCC within the tissue microarray. These findings associate RiboSis-related expression with distinct molecular and inferred immune features in HCC. The clinical relevance of these subtypes for immunotherapy and the functional roles of BOP1 and EXOSC4 require further validation.

## Simple Summary

Hepatocellular carcinoma is a common liver cancer with varied biological and immune features. We analyzed gene expression in individual cells and tumor tissue samples to examine whether genes associated with ribosome production differ between tumors. A hepatocyte population marked by GLUL had the highest ribosome biogenesis-associated score. Tumors separated into two groups with different ribosome-associated gene expression and estimated immune features. In tissue samples, BOP1 and EXOSC4 proteins were more abundant in tumors than in paired adjacent tissues. These findings describe associations that could help guide further research. The immune analyses were computational and do not establish how patients respond to treatment.

## 1. Introduction

Hepatocellular carcinoma (HCC), which accounts for more than 80% of primary liver cancers, remains a major global health burden. According to GLOBOCAN 2024, liver cancer was the seventh most commonly diagnosed cancer and the third leading cause of cancer-related death worldwide, with approximately 843,000 new cases and 732,000 deaths in 2024 [1]. Among Chinese PLC patients, hepatitis B virus (HBV) accounts for 75.09% of cases (with an increasing trend), while HBV–hepatitis C virus (HCV) co-infection constitutes 4.98% [2]. First-line treatments for early-stage HCC include percutaneous ablation, tumor resection, and liver transplantation [3]; however, most cases are diagnosed at intermediate-to-advanced stages, where the 5-year survival rate falls below 10% [4]. Immunotherapy offers promise as a systemic option, including neoadjuvant settings, but its efficacy and safety remain under evaluation [5]. Notably, HCC exhibits low immunotherapy sensitivity, with objective response rates of only ~20% to PD-1 checkpoint inhibitor monotherapy [6,7], largely due to complex mechanisms underlying its pathogenesis, progression, and drug resistance [8,9]. A better understanding of tumor-intrinsic biological programs and their relationships with the immune microenvironment may therefore help identify molecular features associated with HCC heterogeneity and potential therapeutic response.

Ribosome biogenesis (RiboSis) is a tightly coordinated process involving RNA polymerases, ribosomal proteins (RPs), and trans-acting factors to produce ribosomes for protein synthesis; its hyperactivation promotes uncontrolled cell proliferation [10,11,12]. Previous studies suggest that HBV and HCV proteins can promote ribosome biogenesis by enhancing rRNA transcription through distinct molecular mechanisms [13,14]. Emerging evidence shows that excessive ribosome synthesis not only drives tumor initiation but also serves as a hallmark of cancer malignancy and prognosis [15,16]. In addition to their canonical roles in ribosome assembly and translation, several ribosomal proteins possess extraribosomal functions involved in cell-cycle regulation, stress responses, and immune signaling [17,18,19]. Ribosomal heterogeneity may also influence the translation of specific subsets of transcripts and thereby alter tumor-associated phenotypes. For example, changes in ribosomal P-stalk composition have been linked to altered HLA class I expression and CD8^+^ T-cell-mediated tumor recognition, suggesting a potential connection between ribosomal regulation and antitumor immunity [20]. Despite accumulating evidence linking dysregulated ribosome biogenesis to cancer progression, the cellular distribution of RiboSis activity within HCC and its relationship with molecular heterogeneity and the immune microenvironment remain incompletely characterized. In particular, it is unclear which HCC cell populations exhibit elevated RiboSis activity, how RiboSis-associated transcriptional programs relate to intercellular communication and immune infiltration, and whether these features can be used to define biologically distinct HCC subtypes.

To address these questions, we integrated single-cell and bulk transcriptomic analyses to characterize RiboSis-associated cellular states and identify candidate RiboSis-related genes in HCC. We further investigated RiboSis-related molecular subtypes with respect to pathway activity, inferred immune-cell infiltration, somatic mutation patterns, predicted drug and immunotherapy responses, and tissue-level protein expression. These analyses were intended to characterize associations between RiboSis and HCC heterogeneity and to generate hypotheses for subsequent experimental and clinical validation.

## 2. Materials and Methods

### 2.1. Data Collection

Single-cell RNA-sequencing data were obtained from the Gene Expression Omnibus (GEO; https://www.ncbi.nlm.nih.gov/geo/ accessed on 30 October 2026; GSE149614, GPL24676) database, including 10 primary HCC and 8 adjacent non-tumor samples. Data were processed using Seurat (version 4.2.0) [21]. Unexpressed genes were removed, and cells with 200–8000 detected genes, fewer than 100,000 unique molecular identifiers (UMIs), and mitochondrial counts accounting for less than 20% of total UMIs were retained. Expression data were normalized using NormalizeData, followed by highly variable gene selection and principal component analysis (PCA). Harmony was used to adjust for sample-associated batch effects. Graph-based clustering was performed using FindClusters with 16 components and a resolution of 0.2, yielding 12 clusters. Uniform manifold approximation and projection (UMAP) was used for visualization. Cluster markers were identified using FindAllMarkers with default parameters, and cell types were annotated using established markers. Cell-type proportions were subsequently calculated.

Bulk gene expression profiles and clinical data were obtained from The Cancer Genome Atlas (TCGA; https://portal.gdc.cancer.gov/) and GEO. TCGA-LIHC expression data in transcripts per million (TPM), clinical information, and single nucleotide variation (SNV) data generated by the VarScan2 variant-calling workflow were retrieved using TCGAbiolinks (version 2.25.0) [22]. The dataset included 374 tumor and 50 non-tumor samples, with complete survival information for 338 tumors. GSE76427 (GPL10558), comprising 115 HCC tumor and 52 adjacent non-tumor samples, was downloaded using GEOquery (version 2.62.2). The merged training cohort contained 489 tumor and 102 non-tumor samples. Dataset-associated batch effects were adjusted using ComBat in the sva package (version 3.52.0) [23], with PCA used to assess variation before and after correction. All data were accessed according to database policies.

### 2.2. Identification of RiboSis-Associated Cells

The 331 RiboSis-related genes were taken from Zang et al. [10]. RiboSis gene-set activity at the single-cell level was quantified using the R package AUCell (version 1.28.0) [24]. AUCell first ranks all genes within each individual cell according to their expression levels and then evaluates the enrichment of RiboSis-related genes among the highly expressed genes in each cell. The RiboSis activity score for each cell was calculated as the area under the recovery curve (area under the curve, AUC). Cells in which RiboSis-related genes were more strongly enriched at the top of the gene-expression ranking exhibited higher AUC values. The AUCell_exploreThresholds function was used to determine the threshold for identifying cells with active RiboSis gene sets, thereby defining cells with relatively high RiboSis activity. Finally, single-cell AUC scores were projected onto the UMAP embedding using the R package ggplot2 (version 3.3.5) to visualize the distribution of RiboSis activity across individual cells.

### 2.3. Pseudotime Trajectory Reconstruction

To explore dynamic gene expression programs driving HCC progression, pseudotime trajectory reconstruction was performed using Monocle 2 (version 2.34.0) [25]. Highly variable and highly expressed genes (dispersion estimate ≥ 1 and average expression ≥ 0.1) were selected for trajectory inference.

### 2.4. Cell–Cell Communication Analysis

Cell–cell communication networks among HCC-associated cell types were inferred using the R package CellChat (version 1.6.1). CellChat objects were constructed from the UMI count matrix (https://www.github.com/sqjin/CellChat accessed on 28 September 2026), and intercellular signaling interactions were predicted using the CellChatDB.human ligand–receptor interaction database with default parameters.

### 2.5. Single-Cell Regulatory Network Inference and Drug Sensitivity Analysisn

Key transcription factors (TFs) were identified through cis-regulatory network analysis using pySCENIC (version 0.11.2) [26]. Regulons, consisting of TFs and their direct target genes, were constructed, and active regulators were determined using the default AUCell threshold. Regulator specificity across cell types was further quantified using the Regulator Specificity Score (RSS). In parallel, the Drug–Gene Interaction Database (DGIdb, https://dgidb.org/) was queried to predict candidate drugs or small-molecule compounds targeting prognostic genes.

### 2.6. Integrated Identification and Functional Annotation of RiboSis-Related Genes

Differentially expressed genes from the single-cell and bulk transcriptomic analyses were intersected with the reference RiboSis-related gene set. The resulting candidates were subjected to Gene Ontology (GO) and Kyoto Encyclopedia of Genes and Genomes (KEGG) enrichment analyses. Feature selection was performed using LASSO regression, random forest, and SVM-RFE. LASSO regression was performed using the glmnet package (Version 4.1-10) (α = 1) with a binomial model and 10-fold cross-validation over 100 candidate λ values. The λ value corresponding to the minimum cross-validation error (λ.min) was selected, and genes with non-zero coefficients were retained. Random forest classification was implemented using the mlr3 framework. Five-fold cross-validation and grid search with log-loss as the optimization metric were used to tune ntree (1–1000; 100-step resolution) and mtry (1 to one less than the number of candidate genes). Feature importance was evaluated using mean decrease in accuracy (MDA) and mean decrease in Gini (MDG), with the top 10 genes from each ranking retained. SVM-RFE was performed using a linear-kernel support vector machine with 10-fold cross-validation and log-loss evaluation. The procedure was repeated across 20 randomly selected seeds (1–10,000; set.seed = 13), and feature selection was based on cross-validation performance. Finally, the genes selected by all three methods were intersected to identify consensus candidate genes.

### 2.7. Molecular Subtype Identification and Downstream Gene Selection

Consensus clustering was performed using the ConsensusClusterPlus package (version 1.58.0) in R based on the expression profiles of the consensus candidate genes in HCC samples from the combined cohort. Candidate cluster numbers (k = 2–6) were evaluated using cumulative distribution function (CDF) curves and consensus matrices to assess clustering stability and separation. Differential expression analysis was subsequently performed between the identified molecular subtypes, and the resulting differentially expressed genes were intersected with the consensus candidate gene set to identify subtype-associated genes. The gene-selection and clustering workflow is summarized in Supplementary Figure S1.

### 2.8. GSEA and GSVA Analysis

GSEA is a computational method that evaluates whether a predefined set of genes exhibits statistically significant and concordant differences between two biological states. In this study, differential gene expression analysis between subtypes defined by RiboSis activity was performed using the R package “limma” (version 3.50.0) to obtain log_2_ fold-change (FC) values [27]. All genes were ranked based on log_2_FC, and GSEA was conducted using the “clusterProfiler” R package (version 4.2.2) with 1000 permutations per gene set. The reference gene set c2.cp.kegg.v7.5.1.symbols, obtained from the Molecular Signatures Database (MSigDB) [28], was used for enrichment analysis. Gene sets with a nominal *p*-value < 0.05 were considered significantly enriched. To further explore biological functional heterogeneity among HCC subtypes, GSVA was applied using the “GSVA” R package (version 1.42.0) based on the same KEGG gene set (c2.cp.kegg.v7.5.1.symbols). The results were visualized using the “pheatmap” R package (version 1.0.12).

### 2.9. ssGSEA-Based Immune Cell Signature Analysis

Immune cell infiltration was quantified using single-sample GSEA (ssGSEA) based on 28 immune cell signatures obtained from the TISIDB database (http://cis.hku.hk/TISIDB/; accessed on 30 October 2026) [29]. Subtype-specific immune infiltration patterns were compared, and correlations between hub gene expression and immune cell abundance were evaluated.

### 2.10. Drug Sensitivity and TMB

The genomic mutation landscape was constructed using mutation and copy number variation data. Somatic mutation profiles across the different subtypes were summarized and visualized using the R package maftools (version 3.3.6) [30]. Drug sensitivity was assessed using half-maximal inhibitory concentration (IC50) data and corresponding gene expression profiles from the Genomics of Drug Sensitivity in Cancer (GDSC, https://www.cancerrxgene.org/) database. Potential therapeutic drug sensitivity in patients with different RiboSis-related HCC subtypes was predicted using the R package oncoPredict (version 0.2) [31].

### 2.11. Immunotherapy Response (TIDE) and Immune Checkpoint Analysis

The Tumor Immune Dysfunction and Exclusion (TIDE) platform (https://cide.ccr.cancer.gov/tide/ accessed on 30 October 2026) was used to indicate potential immunotherapy-associated features. Expression levels of immune checkpoint genes (ICGs) were compared between subtypes [32].

### 2.12. Pan-Cancer Analysis

Hub genes were analyzed across 33 TCGA cancer types to examine their pan-cancer expression, prognostic value, and immune correlations. Differences in hub gene expression between tumor and normal tissues were assessed using the Wilcoxon test. Survival associations were evaluated using univariate Cox regression. Immune infiltration scores were calculated by ssGSEA, and correlations between hub gene expression and immune infiltration were determined.

### 2.13. Tissue Microarray (TMA) and Immunohistochemistry (IHC)

TMA chips were purchased from Shanghai Weiaobio Biotechnology Co., Ltd. (Shanghai, China). Each TMA chip contained 48 cases, including HCC tissues and their paired normal tissues (paracancerous tissues), with each case represented in duplicate. The TMA sections were incubated with primary antibodies against EXOSC4 (polyclonal antibody, 15937-1-AP, Proteintech, Hubei, China) and BOP1 (anti-BOP1 antibody, Abcam, Shanghai, China). Quantitative analysis of the number of positively stained cells and their staining intensity on each section was performed using APTime image analysis software (Shanghai, China) to obtain the histochemical score (H-score). The H-score was calculated as H-score = Σ (Pi × i) = (percentage of weakly stained cells × 1) + (percentage of moderately stained cells × 2) + (percentage of strongly stained cells × 3), where Pi represents the percentage of positively stained area or cells, and i denotes the staining intensity. A higher H-score indicates a stronger overall positive staining intensity.

### 2.14. Statistical Analysis

This study was statistically analyzed using R software v4.1.2. Spearman correlation test was used to infer the correlation between the two parameters. The Wilcoxon test was used to compare differences between two groups, and the Kruskal–Wallis test was used to compare differences between three or more groups. A two-sided p-value of less than 0.05 was considered statistically significant.

## 3. Results

### 3.1. Single-Cell Clustering, Annotation, and Identification of RiboSis-Related Cells

**Figure 1:**
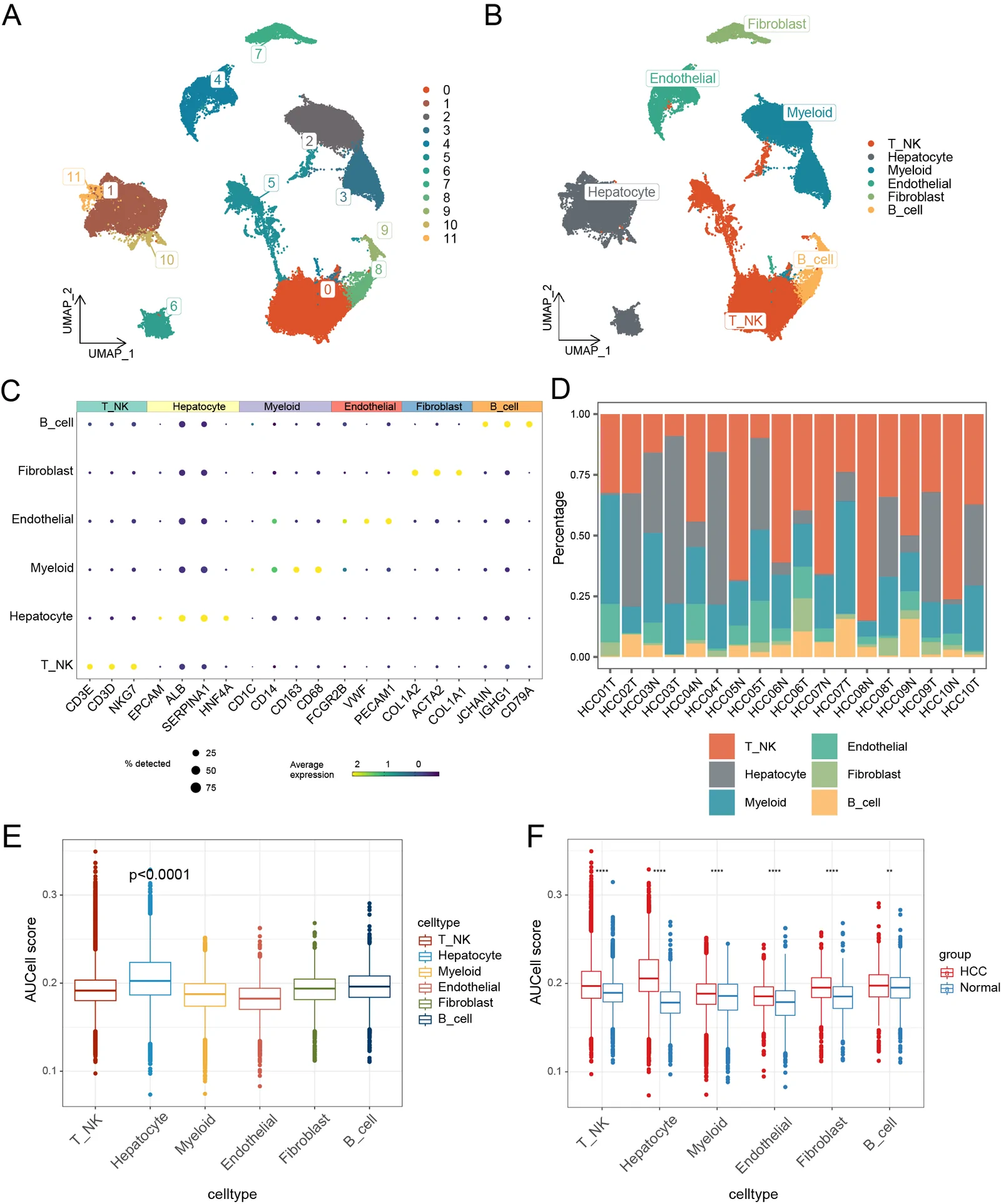
Identification of cellular subpopulations in HCC (GSE149614). (**A**) UMAP visualization of cell clusters; (**B**) Annotation of six major cell types; (**C**) Dot plot of cluster-specific marker genes; (**D**) Stacked bar chart of cell type proportions across samples; (**E**) RiboSis activity across cell types; (**F**) RiboSis activity in HCC vs. control groups by cell type. ** *p* < 0.01, **** *p* < 0.0001.

We obtained 59,578 cells from single-cell transcriptomic data of 18 samples, with even distribution and no significant batch effects. Clustering identified 12 distinct clusters (Figure 1A), which were annotated into six major cell types based on established biomarkers (Figure 1C, Table S2): T/NK cells, B cells, hepatocytes, endothelial cells, myeloid cells, and fibroblasts (Figure 1B). Among these, T/NK cells, myeloid cells, and hepatocytes were the most abundant (Figure 1D).

To identify the major cellular source of RiboSis in HCC, we classified cells into high (AUCell score > 0.21032913) or low (AUCell score ≤ 0.21032913) RiboSis activity, yielding 11,390 high-activity cells. Hepatocytes showed significantly elevated activity upon HCC onset (Figure 1F) and higher activity than other cell types (Figure 1E).

**Figure 2:**
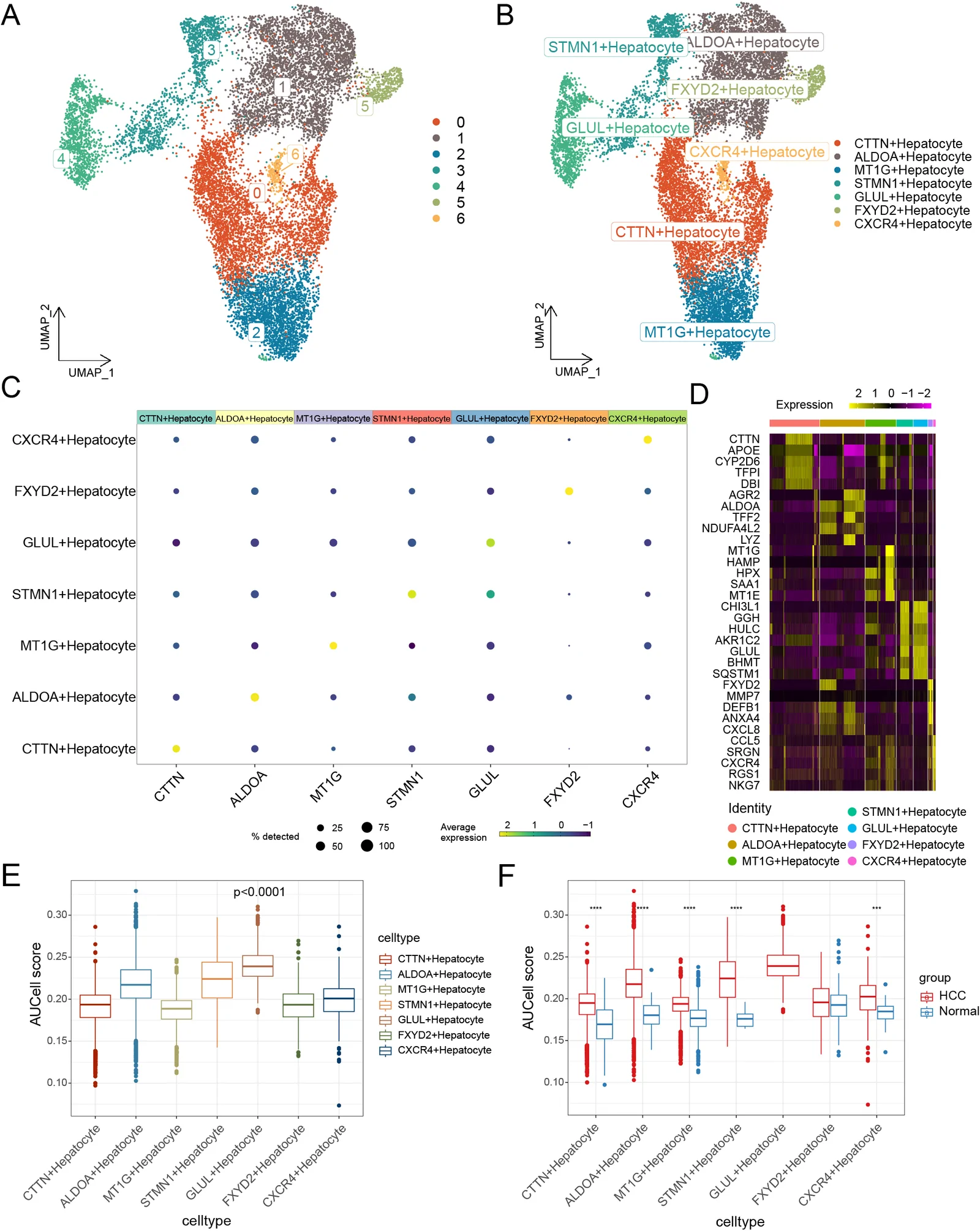
Identification and characterization of hepatocyte subclusters based on RiboSis-related gene-set scores. (**A**) UMAP plot of hepatocyte subclusters. (**B**) UMAP plot showing the annotation and distribution of the seven hepatocyte subclusters, named according to their representative marker genes: CXCR4^+^, FXYD2^+^, GLUL^+^, STMN1^+^, MT1G^+^, ALDOA^+^, and CTTN^+^ hepatocytes. The “^+^” designation indicates expression of the corresponding marker gene. (**C**) Dot plot illustrating marker genes specifically expressed in each subcluster. (**D**) Heatmap of differentially expressed genes among hepatocyte subclusters. (**E**) Box plots comparing AUCell scores for the RiboSis-related gene set across the seven hepatocyte subclusters. (**F**) Box plots comparing AUCell scores for the RiboSis-related gene set between HCC and control samples within each hepatocyte subcluster. *** *p* < 0.001, **** *p* < 0.0001.

Reclustering of hepatocytes revealed seven subclusters (Figure 2A), annotated as CXCR4^+^, FXYD2^+^, GLUL^+^, STMN1^+^, MT1G^+^, ALDOA^+^, and CTTN^+^ hepatocytes based on signature genes (Figure 2B–D, Table S3). GLUL^+^ hepatocytes exhibited the highest AUCell score for the RiboSis-related gene set among the hepatocyte subclusters (Figure 2E), which was specifically enriched in the HCC group, and were therefore selected for subsequent analyses.

### 3.2. Pseudotime Analysis

**Figure 3:**
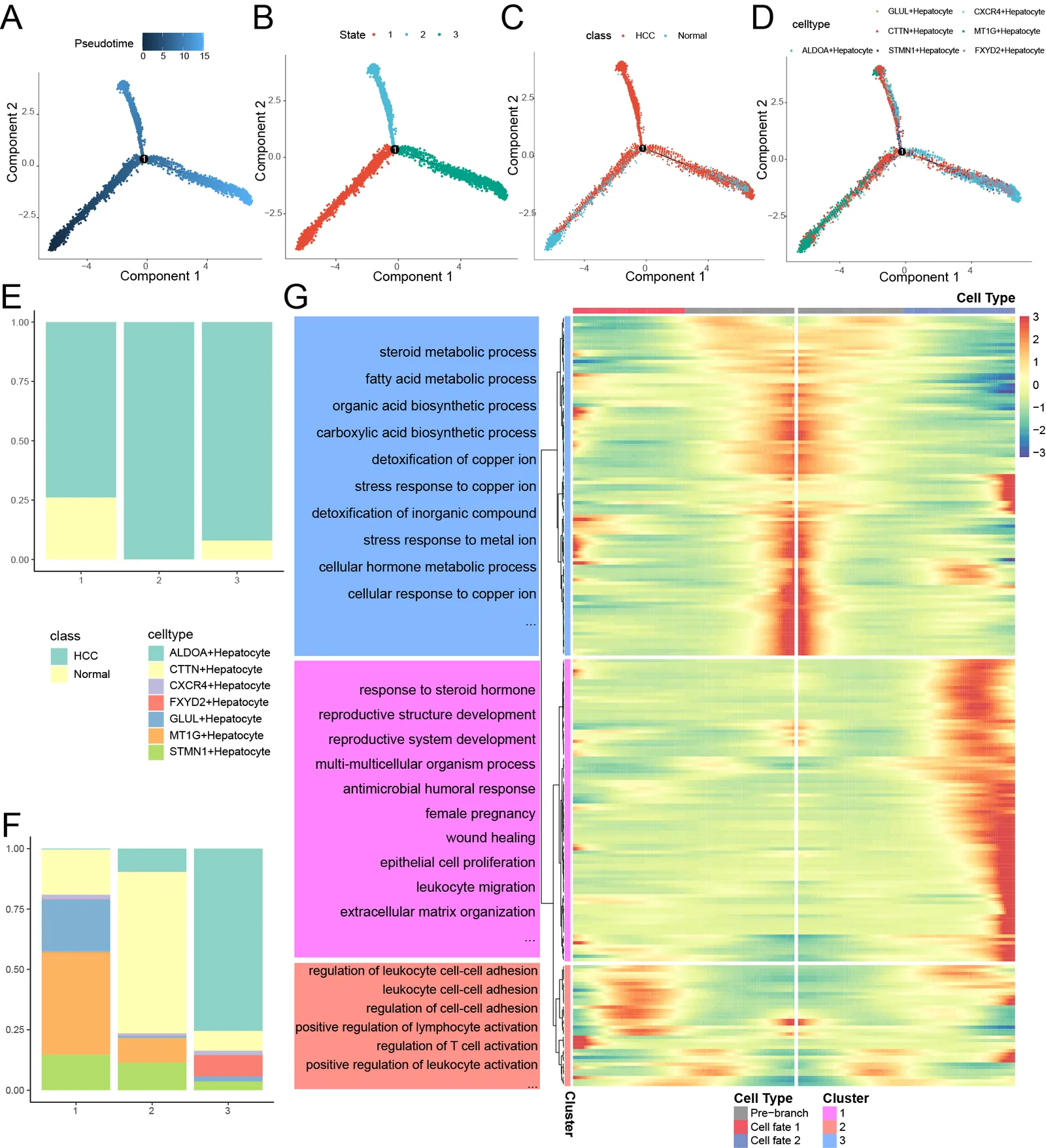
Pseudotemporal trajectory analysis reveals hepatocyte differentiation dynamics and suggests functional disparities in HCC. (**A**) Pseudotime trajectory showing the direction of differentiation, with a color gradient from dark to light blue indicating progression from start to end points. (**B**) Three distinct cell states identified along the pseudotemporal trajectory of hepatocytes by Monocle2. (**C**) Distribution of hepatocytes from HCC and control groups across the pseudotime trajectory. (**D**) Distribution of hepatocyte subclusters along the pseudotemporal trajectory. (**E**) Stacked bar chart showing the proportion of cells in each state within HCC and control groups. (**F**) Stacked bar chart displaying the composition of hepatocyte subclusters in each state. (**G**) Heatmap of differentially expressed genes across branching cell fates. Significantly enriched GO terms for selected gene clusters are shown on the left.

To investigate functional changes during hepatocyte differentiation and their role in HCC progression, we reconstructed a pseudotemporal trajectory. The trajectory revealed distinct transcriptional states (Figure 3A), with hepatocytes diverging at one major branching point into three differentiation states (Figure 3B). HCC samples were predominantly localized in State 1 and State 3 (Figure 3C,E). Subcluster analysis showed that CTTN^+^ hepatocytes concentrated in State 2, whereas GLUL^+^ hepatocytes were predominantly in State 1 (Figure 3D,F).

From the branch point (Node 1), three lineages emerged: State 1 (early phase) enriched in steroid and fatty acid metabolism; Cell Fate 1 (State 2) enriched in leukocyte adhesion and lymphocyte activation; and Cell Fate 2 (State 3) enriched in steroid hormone response and reproductive system development (Figure 3G, Table S4).

### 3.3. Cell Communication Analysis

**Figure 4:**
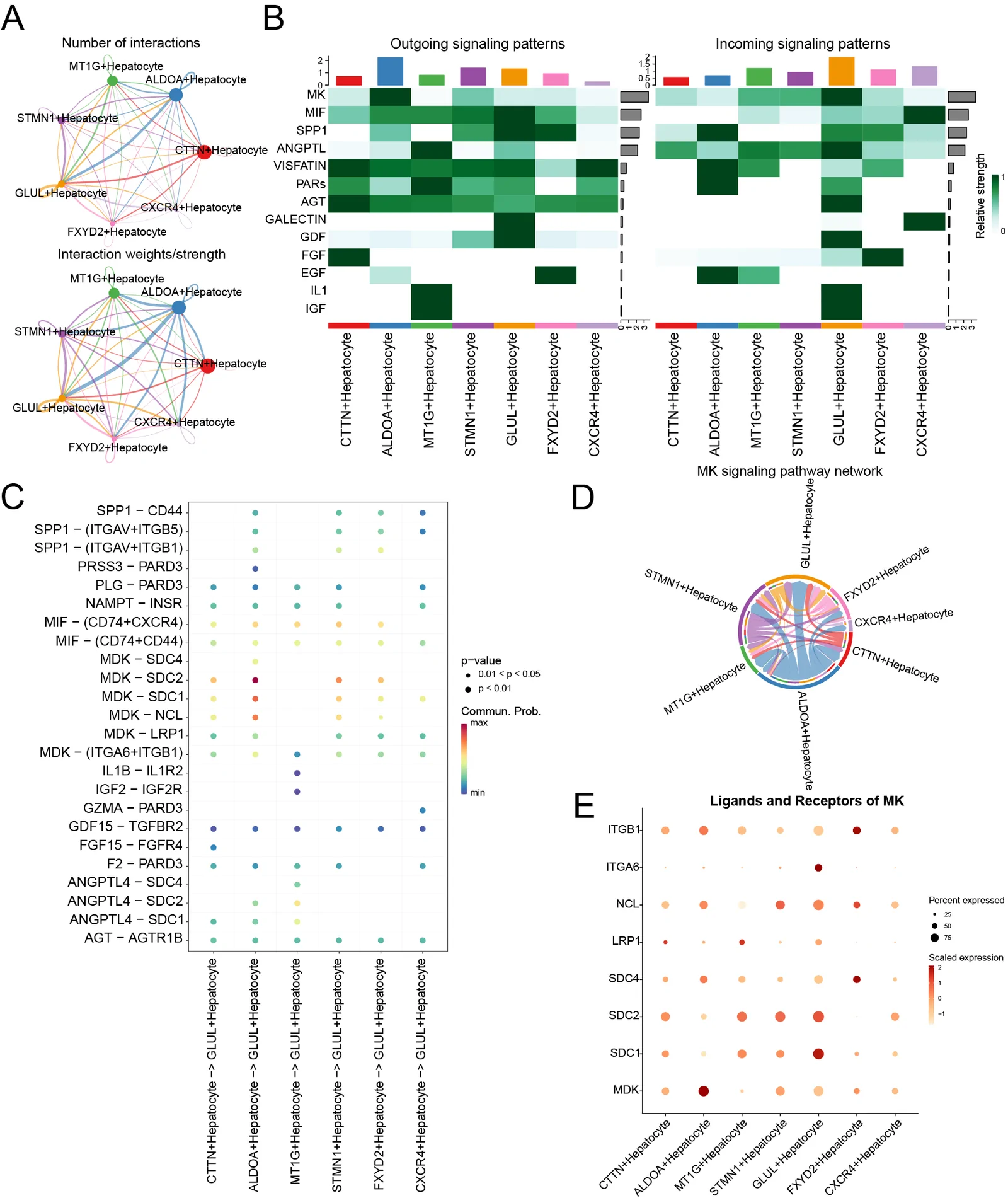
Analysis of cell–cell communication among hepatocyte subclusters in HCC. (**A**) Network plots showing the number and strength of intercellular interactions. (**B**) Heatmaps visualizing potential incoming or outgoing signaling pathways between cell clusters. (**C**) Dot plots visualizing potential incoming or outgoing ligand–receptor pairs between cell clusters. (**D**) Chord diagrams visualizing potential incoming or outgoing signaling pairs between cell clusters. (**E**) Expression distribution of ligands and receptors in the MK signaling pathway.

To elucidate intercellular interactions in the HCC tumor immune microenvironment, we performed cell–cell communication analysis across all identified cell types (Figure 4A). Among hepatocyte subclusters, ALDOA^+^ hepatocytes primarily acted as signal senders, whereas GLUL^+^ hepatocytes served as major signal receivers (Figure 4B). Key signaling pathways included MK, MIF, SPP1, ANGPTL, and VISFATIN. The most prominent interaction was mediated by the MDK–SDC2 axis between ALDOA^+^ and GLUL^+^ hepatocytes (Figure 4C,D). Specifically, MK pathway ligands were highly expressed in ALDOA^+^ hepatocytes, and the receptor SDC2 in GLUL^+^ hepatocytes (Figure 4E). These findings clarify potential communication mechanisms among hepatocyte subpopulations in HCC.

### 3.4. Single-Cell Regulatory Network Analysis and Drug Prediction

**Figure 5:**
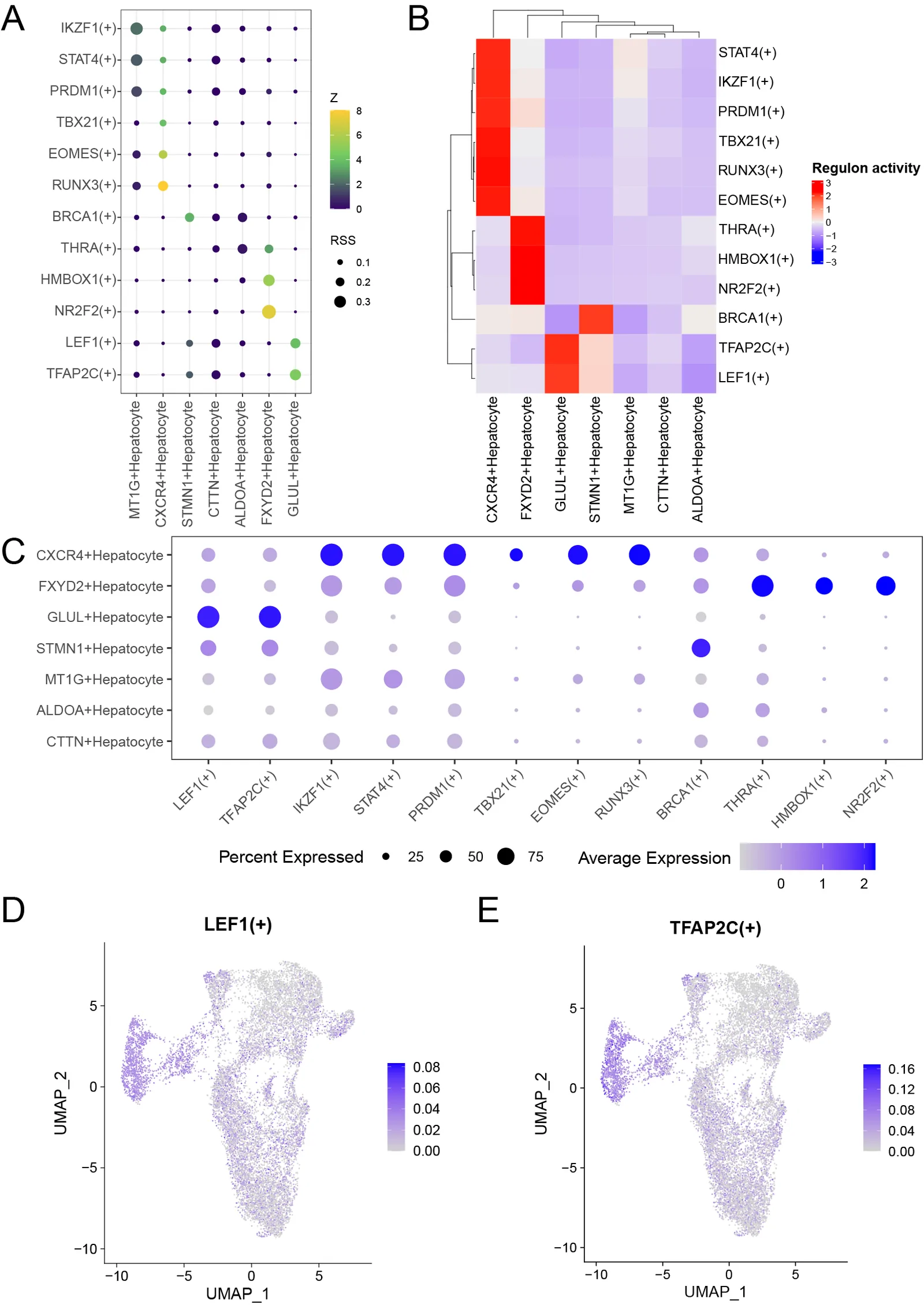
Single-cell regulatory network analysis. (**A**) Dot plot displaying cell type–specific TFs. (**B**) Heatmap of cell type–specific TFs. (**C**) Dot plot showing expression levels of selected TFs across cell clusters. (**D**) UMAP visualization of LEF1(+) expression in hepatocytes. (**E**) UMAP visualization of TFAP2C(+) expression in hepatocytes.

TFs potentially regulating hepatocyte subpopulations in HCC, we performed SCENIC analysis. Bubble plots and heatmaps were used to visualize TF specificity across cell types (Figure 5A,B) and their expression levels (Figure 5C), respectively. Among these, LEF1 and TFAP2C were found to be highly and specifically expressed in GLUL^+^ hepatocytes. Subsequent UMAP visualization confirmed that both LEF1 (Figure 5D) and TFAP2C (Figure 5E) were exclusively expressed within the GLUL^+^ hepatocyte subpopulation. Drug interaction prediction for LEF1 and TFAP2C was conducted using the DGIdb database; however, no known targetable compounds or interacting drugs were identified for these TFs.

### 3.5. Screening and Enrichment of RiboSis–Related Genes in HCC

**Figure 6:**
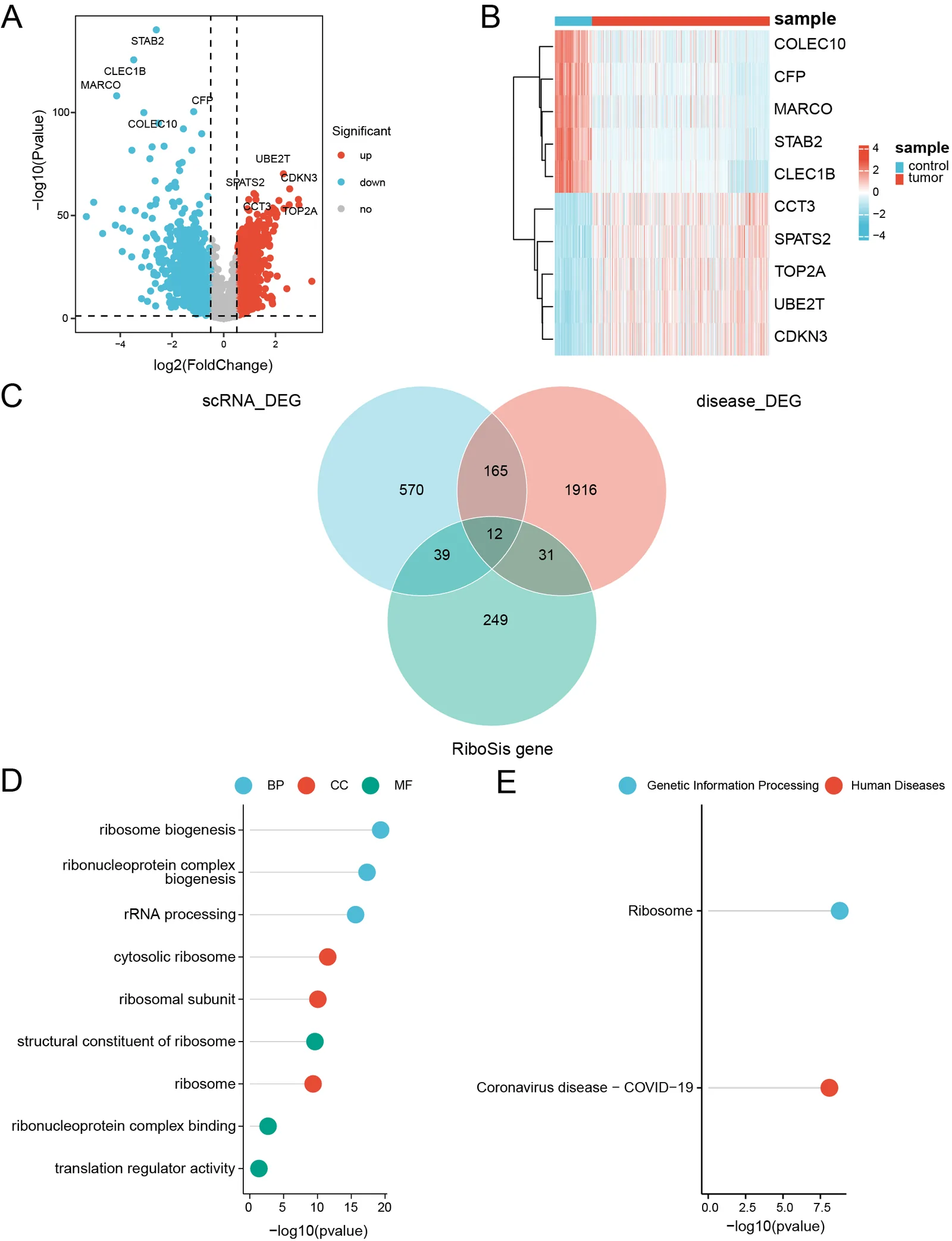
Identification and functional enrichment of key genes. (**A**) Volcano plot of the top five up- and down-regulated genes in HCC versus control groups from transcriptomic data. (**B**) Heatmap displaying the most significantly up- and down-regulated genes between HCC and control groups. (**C**) Venn diagram illustrating the selection of hub genes. (**D**) GO enrichment analysis of the hub genes. (**E**) KEGG pathway enrichment analysis of the hub genes. Bulk transcriptomic data were obtained from TCGA-LIHC and GEO dataset GSE76427, while the single-cell transcriptomic data were obtained from GEO dataset GSE149614.

To identify genes associated with RiboSis activity in HCC, we first performed differential gene expression analysis between GLUL^+^ hepatocytes and other hepatocyte subclusters, yielding 786 differentially expressed genes (DEGs) (Table S5). Additionally, we identified 2124 DEGs by comparing transcriptomic data from HCC samples against normal controls (Figure 6A,B, Table S6), which exhibited statistically significant differences and were considered potential regulators of RiboSis. By intersecting these two DEG sets with a reference list of 331 RiboSis–related genes, we identified 12 overlapping genes (RPS14, RPL27, BOP1, NHP2, EXOSC4, RPL35A, RPL7A, AATF, EIF4A3, RPS16, RPS6, RPSA) (Figure 6C, Table S7). These genes are proposed to be related to RiboSis, specifically expressed in GLUL^+^ hepatocytes, and potentially involved in HCC pathogenesis, thus selected for subsequent analyses.

Functional enrichment analysis revealed that these 12 genes were primarily associated with RiboSis such as “ribosome biogenesis”, “ribonucleoprotein complex biogenesis”, and “rRNA processing” (Figure 6D, Table S8). KEGG pathway analysis further indicated significant enrichment in the “Ribosome” pathway and “Coronavirus disease—COVID-19” (Figure 6E, Table S9).

### 3.6. Screening of Hub Genes Using Machine Learning Algorithms

Based on the 12 previously identified candidate genes, three machine learning algorithms (SVM-RFE, LASSO regression, and random forest) were applied for feature selection. LASSO regression identified 10 candidate genes (Figure S2A,B). (Figure S2A,B). The random forest algorithm, evaluated by MDA and MDG, selected the top 9 genes with the highest feature importance (Figure S2C,D). With the SVM-RFE algorithm, 5 key genes were screened (Figure S2E,F). By intersecting the results from these three methods, we ultimately identified 4 consensus genes—BOP1, EXOSC4, NHP2, and RPL27—as hub genes for subsequent analysis (Figure S2G).

### 3.7. Stratification and Pathway Functional Analysis of HCC Patients Based on Hub Genes

Based on hub gene expression, consensus clustering was performed on HCC samples in the training set. With k = 2 selected as the optimal cluster number, samples were divided into Subtype1 and Subtype 2, showing distinct expression patterns of RiboSis-related genes (Figure S3A,B). Among the 338 TCGA-LIHC patients with available clinical records and corresponding subtype assignments, 167 were classified as Subtype 1 and 171 as Subtype 2. The distribution of tumor stages in the two subtypes was compared (Table S10). Differential expression analysis between subtypes, followed by intersection with machine learning-screened key genes (Table S11), identified two hub genes: BOP1 and EXOSC4, both significantly upregulated in Subtype 2 (Figure S3C). ROC analysis confirmed their strong discriminatory power (AUC: 0.8794 for BOP1, 0.8563 for EXOSC4; Figure S3D).

**Figure 7:**
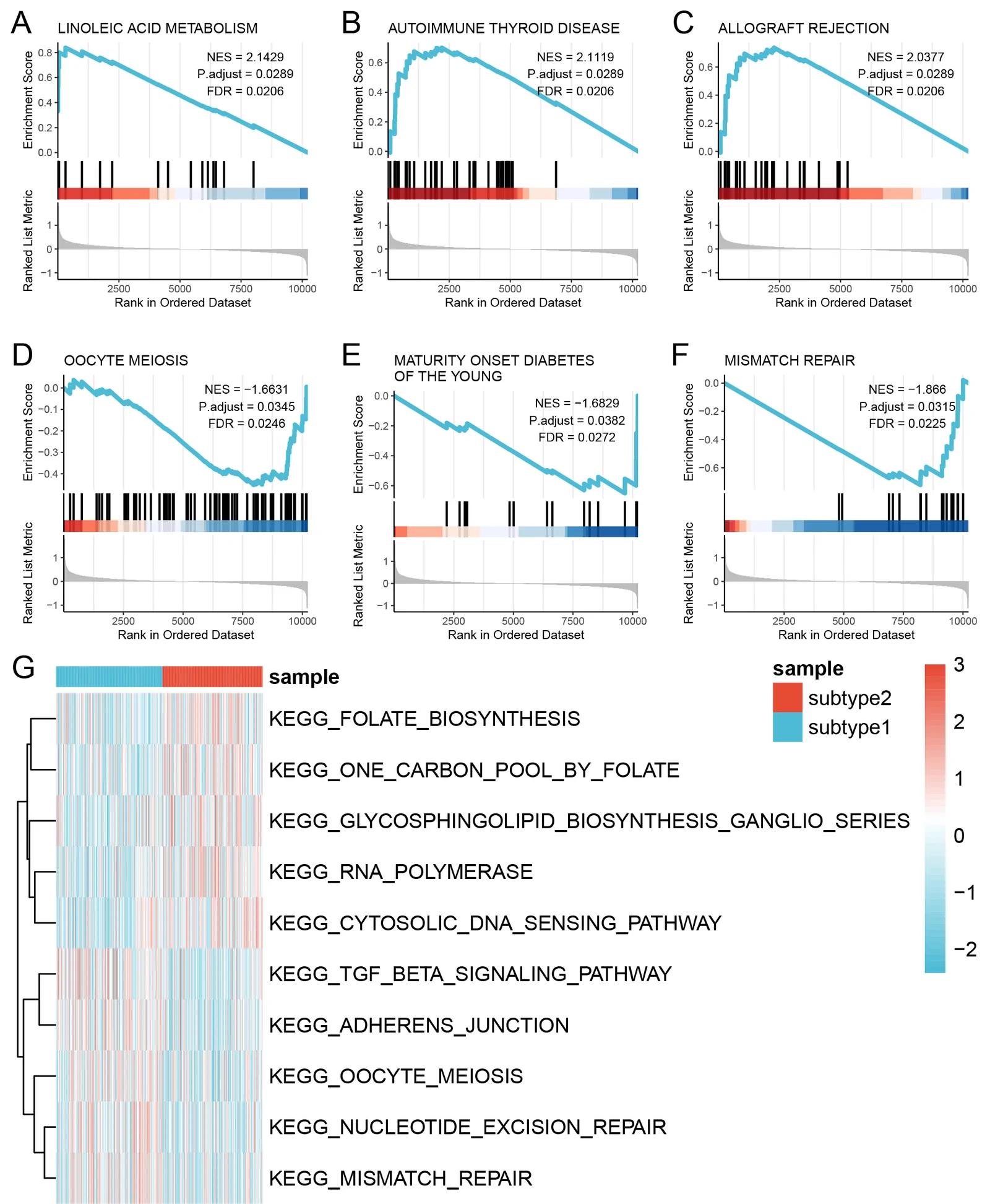
GSEA and GSVA enrichment analysis between RiboSis–related subtypes. (**A**–**F**) Enrichment plots showing significantly enriched KEGG pathways: (**A**) Linoleic acid metabolism, (**B**) Autoimmune thyroid disease, (**C**) Allograft rejection, (**D**) Oocyte meiosis, (**E**) Maturity onset diabetes of the young, (**F**) Mismatch repair. (**G**) Heatmap displaying the top differentially enriched pathways between subtypes based on GSVA. NES: normalized enrichment score. Significantly enriched gene sets were defined as those with *p* < 0.05 and FDR < 0.05. Source data for the bulk transcriptomic analyses were obtained from TCGA-LIHC and GEO dataset GSE76427.

GSEA revealed that Subtype 2 was significantly enriched in linoleic acid metabolism, autoimmune thyroid disease, and allograft rejection (NES > 2.0, adjusted *p* < 0.03), whereas Subtype1 was enriched in oocyte meiosis, maturity onset diabetes of the young, and mismatch repair (NES < −1.66, adjusted *p* < 0.04) (Figure 7A–F, Table S12). GSVA further identified the top five differentially activated pathways between subtypes (Figure 7G, Table S13).

### 3.8. Immune Infiltration Analysis

**Figure 8:**
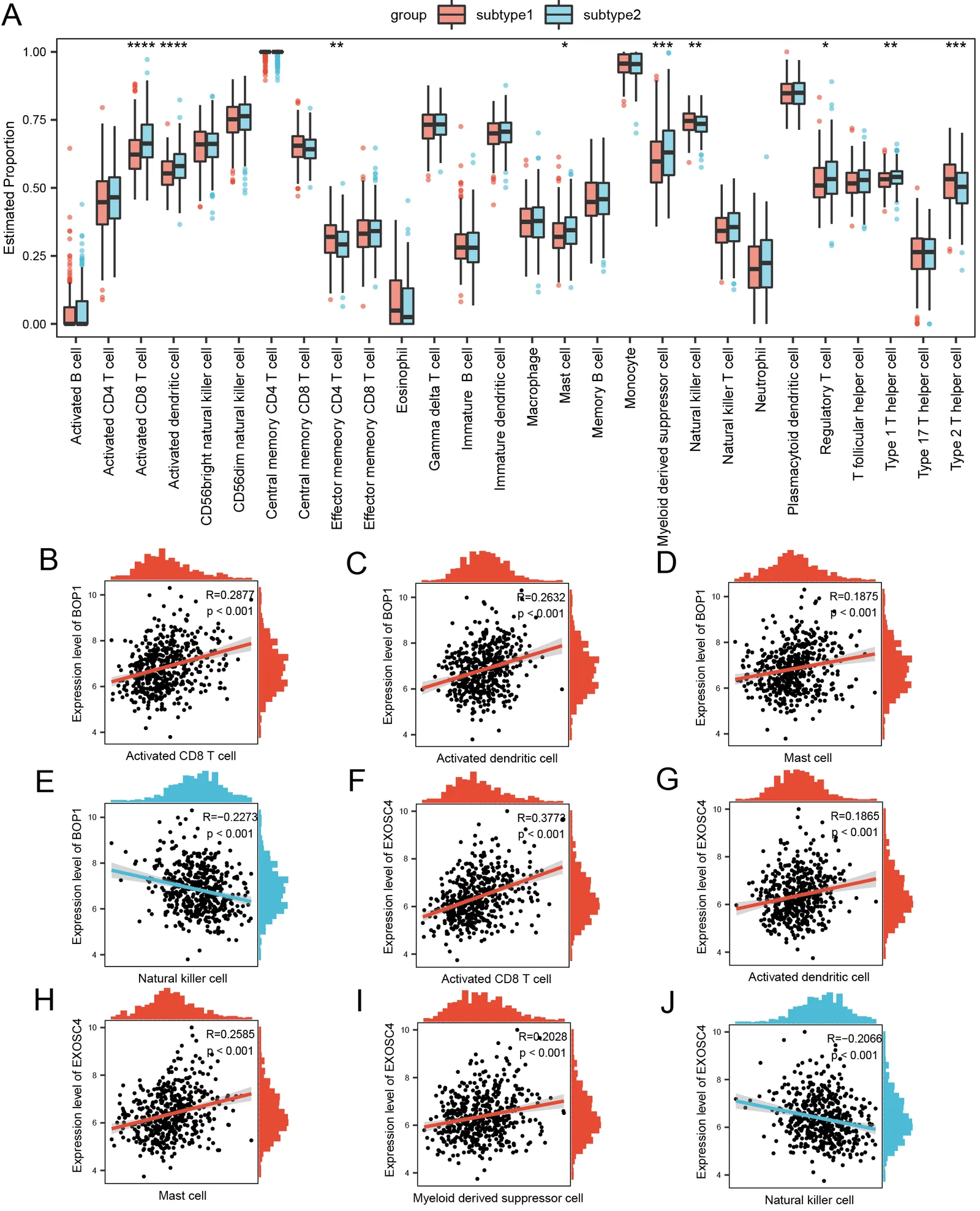
Analysis of immune infiltration differences between RiboSis–related subtypes. (**A**) Box plots showing the estimated proportions of immune cells across subtypes. (**B**–**J**) Scatter plots displaying correlation between hub genes (BOP1 and EXOSC4) and infiltrating immune cells. Source data for the bulk transcriptomic analyses were obtained from TCGA-LIHC and GEO dataset GSE76427. * *p* < 0.05, ** *p* < 0.01, *** *p* < 0.001, **** *p* < 0.0001.

Quantification of 28 immune cell types using ssGSEA (Figure S4A, Table S14) revealed significant positive correlations among most immune cells (Figure S4B). Except for activated B cells, activated CD4^+^ T cells, CD56^bright^ NK cells, γδ T cells, and Th17 cells, most other subsets—including activated CD8^+^ T cells, activated dendritic cells (DCs), and myeloid-derived suppressor cells (MDSCs)—showed significant differences between RiboSis-related subtypes (*p* < 0.05; Figure 8A). Correlation analysis with hub genes showed that BOP1 expression was positively correlated with activated CD8^+^ T cells (R = 0.288, *p* < 0.001), activated DCs (R = 0.263, *p* < 0.001), and mast cells (R = 0.188, *p* < 0.001), and negatively correlated with NK cells (R = −0.227, *p* < 0.001). EXOSC4 was positively correlated with activated CD8^+^ T cells (R = 0.377, *p* < 0.001), activated DCs (R = 0.187, *p* < 0.001), mast cells (R = 0.259, *p* < 0.001), and MDSCs (R = 0.203, *p* < 0.001), and negatively correlated with NK cells (R = −0.207, *p* < 0.001) (Figure 8B–J).

### 3.9. Inter-Subtype Drug Sensitivity Analysis and TMB Analysis

TMB analysis revealed TP53 as the most frequently mutated gene (Figure S5A), with a significantly higher mutation rate in Subtype 2 than Subtype1 (Figure S5B). Drug sensitivity analysis (Table S15) indicated that Subtype1 was more sensitive to Daporinad, BI-2536, and Sepantronium bromide, whereas Subtype 2 was more sensitive to AZD7762, AZD8055, BMS-754807, Rapamycin, and Vinorelbine (Figure S5C–K).

### 3.10. Immune Checkpoint Analysis

TIDE analysis (Table S16) showed that Subtype 1 had higher overall TIDE and Exclusion scores but a lower Dysfunction score than Subtype 2 (Figure S6A). A greater proportion of Subtype 2 samples were classified as potential responders by the TIDE model (Figure S6B). The expression of several immune checkpoint genes, including TNFRSF4, SIGLEC10, and HAVCR2, also differed between subtypes (Figure S6C). Together, these computational findings indicate distinct immune-related profiles.

### 3.11. Relationship Between Pan-Cancer Overall Survival and Hub Genes

To explore the associations between hub gene expression and overall survival (OS), univariate Cox regression analyses were performed across 33 cancer types. Univariate Cox regression analysis across 33 cancer types revealed that high expression of EXOSC4 was significantly associated with OS in 10 cancers. Specifically, it functioned as a risk factor in adrenocortical carcinoma (ACC), uveal melanoma (UVM), and acute myeloid leukemia (LAML), and as a protective factor in lung squamous cell carcinoma (LUSC) and lower-grade glioma (LGG) (Figure S6A). Similarly, BOP1 expression was significantly correlated with OS in 19 cancer types, acting as a risk factor in liver hepatocellular carcinoma (LIHC), ACC, cervical squamous cell carcinoma (CESC), and hematological malignancy (HMSC), and as a protective factor in cholangiocarcinoma (CHOL), LGG, and breast cancer (BRCA) (Figure S7B). These findings represent univariate survival associations and do not establish independent prognostic value.

### 3.12. Immunohistochemical Analysis

**Figure 9:**
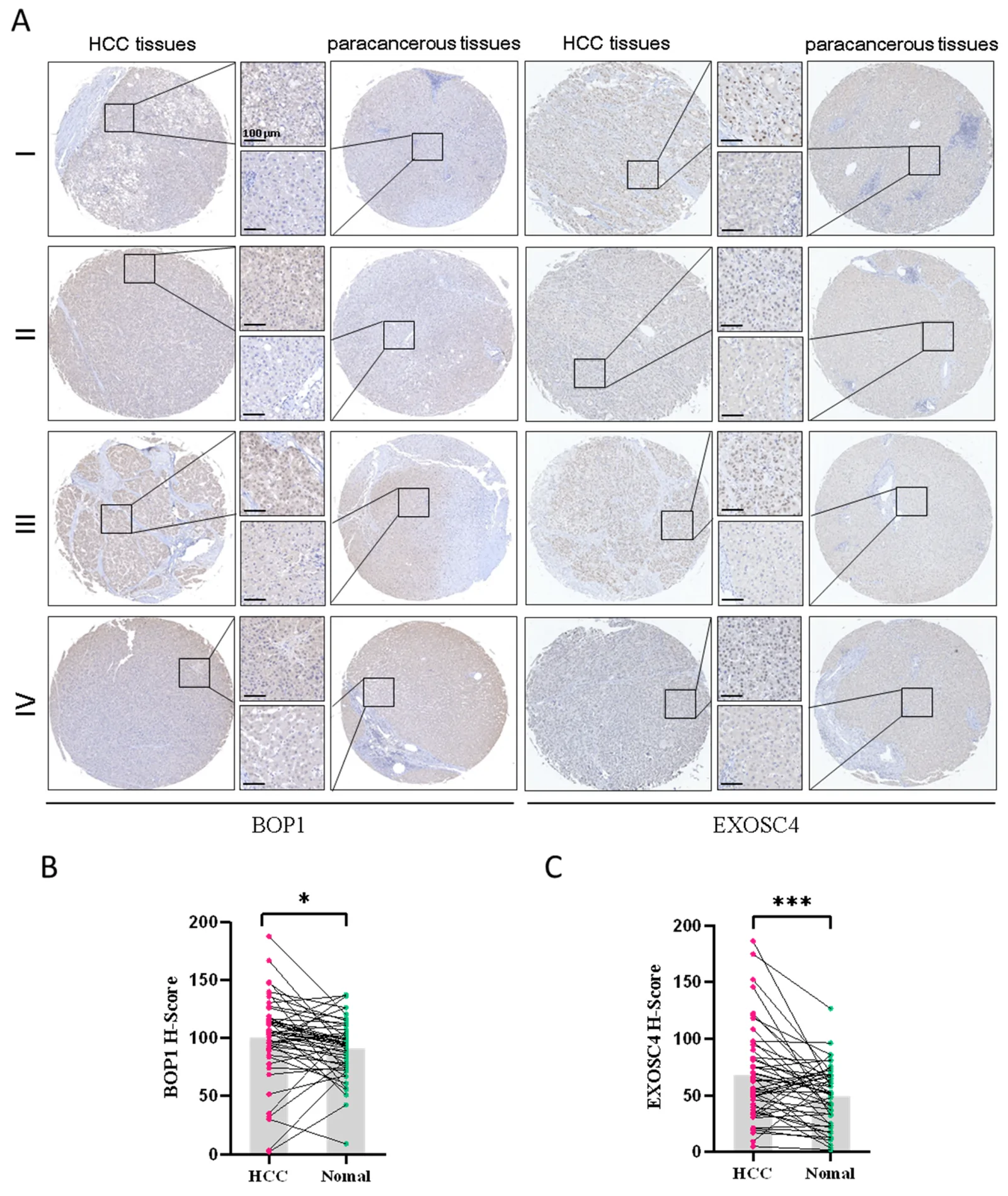
Immunohistochemical analysis of TMA. (**A**) Expression of BOP1 and EXOSC4 in tumor and paired adjacent paracancerous tissues from HCC patients with TNM stage I–IV. (**B**) Quantification of BOP1 expression. (**C**) Quantification of EXOSC4 expression. (“Normal” refers to paired adjacent paracancerous tissues). * *p* < 0.05, *** *p* < 0.001.

We evaluated the expression of these two hub genes on HCC TMA by IHC staining. Representative IHC images of tumor tissues and paired adjacent paracancerous tissues from HCC patients, as well as the corresponding histological grades of the tumors, are shown in Figure 9. H-score analysis demonstrated that BOP1 and EXOSC4 were significantly overexpressed in HCC tissues compared with adjacent paracancerous tissues. Subsequently, we analyzed the clinicopathological characteristics of the patients included in the TMA (Table 1 and Table 2). The IHC H-scores of EXOSC4 were significantly lower in HBV-related HCC patients than in HCC patients without hepatitis virus infection (*p* < 0.001), whereas the expression levels of BOP1 and EXOSC4 were not associated with age, sex, tumor size, AFP level, or other examined clinical parameters.

## 4. Discussion

HCC is the predominant histopathological type of PLC, and its high incidence and recurrence rates impose a significant burden on global public health [2]. In this study, we employed an integrated bioinformatics approach to identify RiboSis–related genes implicated in HCC, leveraging single-cell RNA sequencing and bulk transcriptomic data from public databases including TCGA and GEO. We further defined RiboSis-related molecular subtypes and examined their associations with pathway activity, immune-cell infiltration, somatic mutation patterns, predicted therapeutic response, and tissue-level protein expression.

Ribosome biogenesis involves coordinated synthesis and assembly of ribosomal components [12,33,34]. In our study, reclustering analysis revealed that ribosome biogenesis activity was significantly elevated in GLUL^+^ hepatocytes, which express the glutamine synthetase (GS) gene, compared with other hepatocyte subclusters, and this feature was specific to the HCC group. Previous studies have demonstrated that oncogenic MYC and mTORC1 signaling can promote ribosome biogenesis, metabolic reprogramming, and cell-cycle progression, thereby supporting malignant growth [35,36]. However, the RiboSis score used in this study was derived from transcriptomic gene-set activity and therefore does not directly measure ribosome production or translational output. Similarly, pseudotime trajectories and inferred MDK–SDC2 interactions represent computationally inferred relationships rather than direct evidence of lineage transitions or functional intercellular signaling. Our analyses do not establish whether RiboSis-associated transcriptional changes regulate MDK expression or secretion, or whether the predicted MDK–SDC2 interaction influences T-cell or NK-cell recruitment or function. Experimental perturbation studies will therefore be required to determine the directionality and functional consequences of these associations.

Beyond their canonical roles in ribosome assembly and protein synthesis, selected RPs have been implicated in HCC progression through extra-ribosomal functions, including p53 signaling, cell-cycle regulation, apoptosis, angiogenesis, and epithelial–mesenchymal transition [37]. In our study, seven RP genes (RPS14, RPL27, RPL35A, RPL7A, RPS16, RPS6, and RPSA) were identified among the 12 RiboSis-associated candidate genes. These findings highlight the representation of RP-related genes in the identified transcriptional program. However, their individual functional contributions to HCC progression and immune remodeling were not investigated in the present study and warrant further functional validation. Consensus clustering further stratified HCC samples into two RiboSis-related molecular subtypes with distinct transcriptional profiles. Among the candidate genes identified through the integrated analysis, BOP1 and EXOSC4 emerged as hub genes and showed higher expression in Subtype 2. BOP1 is a nucleolar protein involved in ribosome biogenesis and ribosomal RNA processing. Previous studies have implicated BOP1 in HCC invasion and migration through promotion of epithelial–mesenchymal transition and cytoskeletal remodeling [36]. BOP1 has also been reported to be associated with adverse clinical features and poor prognosis in HCC [34]. EXOSC4 is one of the core structural subunits of the RNA exosome complex, drives tumor progression by maintaining oncogenic RNA stability, and has become an emerging prognostic marker and potential therapeutic target [38,39].

Pathway analyses revealed significant enrichment of linoleic acid metabolism, autoimmune thyroid disease(AITD), and allograft rejection gene sets in Subtype 2. These enrichment results should be interpreted as differences in the expression of genes annotated to these pathways rather than evidence that the corresponding disease processes themselves occur in HCC. Altered linoleic acid metabolism may nevertheless be biologically relevant to HCC. Recent experimental evidence suggests that HCC cells can compete with T cells for linoleic acid, thereby impairing T-cell function and promoting tumor progression [40]. The enrichment of immune-related gene sets such as autoimmune thyroid disease and allograft rejection may similarly reflect differences in immune activation, inflammatory signaling, or antigen-processing pathways between the two subtypes rather than disease-specific processes. Consistent with this interpretation, immune infiltration analysis revealed distinct immune-cell signatures between the RiboSis-related subtypes. BOP1 and EXOSC4 expression was positively correlated with inferred infiltration of activated CD8^+^ T cells, activated dendritic cells, and mast cells, and negatively correlated with NK-cell infiltration. These associations suggest that RiboSis-related transcriptional programs may be linked to the immune composition of the HCC microenvironment. MHC class I molecules are central to antigen presentation to CD8^+^ T cells, whereas NK cells are sensitive to alterations in MHC-I expression through missing-self recognition [41,42]. However, the present study did not directly measure tumor-cell MHC-I abundance, KIR/NKG2A signaling, or immune-cell function. Therefore, the observed associations cannot establish that BOP1, EXOSC4, or RiboSis activity directly regulates CD8^+^ T-cell or NK-cell infiltration through MHC-I-dependent mechanisms. These possibilities should be regarded as mechanistic hypotheses for future investigation.

Moreover, Subtype 2 showed a higher tumor mutational burden than Subtype 1. Although higher TMB has been associated with improved outcomes following immune checkpoint inhibition in some cancer types [43], this association does not establish treatment benefit in the present HCC cohort. TIDE analysis showed lower overall TIDE and Exclusion scores but a higher Dysfunction score in Subtype 2, indicating differences in computationally inferred immune escape features [32]. Together with the observed differences in immune cell signatures, these findings suggest that the two subtypes have distinct immune profiles that warrant investigation in the context of immunotherapy. Because no immunotherapy-treated cohort or observed response endpoint was analyzed, whether these subtypes differ in clinical benefit from immune checkpoint inhibitors remains unknown. In hepatocellular carcinoma, the ribosome biogenesis-associated factor RRS1 has been reported to correlate with the expression of immune checkpoint genes, including PDCD1 and CD274 [44]. However, a direct regulatory relationship between RiboSis and PD-1 was not established in the present study, and further investigation is needed to clarify this potential association.

Interestingly, EXOSC4 expression was lower in HBV-associated HCC than in non-viral HCC samples in our TMA cohort. Previous studies have indicated that RNA exosome components participate in RNA surveillance and may contribute to antiviral responses [45,46]. However, the basis for this difference remains unclear, and our findings do not establish a causal link between EXOSC4 expression and HBV replication, persistence, or HBV-associated hepatocarcinogenesis. Further studies incorporating well-characterized clinical cohorts and functional validation are warranted to clarify the potential association between EXOSC4 expression and HCC etiology.

Several limitations should be considered. First, this study mainly relied on retrospective public datasets, and prospective multicenter cohorts are required to validate the clinical applicability of the proposed RiboSis-associated classification. Although cross-validation was employed during machine-learning-based feature selection, independent external validation of the selected genes was not performed. Therefore, their robustness and generalizability require further evaluation. Kaplan–Meier survival analysis was not performed between the two molecular subtypes; therefore, the prognostic stratification value of this classification remains to be established. Multivariable Cox regression incorporating clinical covariates was not performed for BOP1 and EXOSC4; therefore, their independent prognostic value remains to be established. AFP levels and liver disease etiology were unavailable in the clinical records of the TCGA-LIHC patients included in the subtype analysis, precluding further evaluation of their associations with the identified molecular subtypes. In addition, survival follow-up data were unavailable for the TMA cohort, precluding assessment of the associations between BOP1/EXOSC4 expression and OS or DFS. Second, although tissue microarray analysis confirmed the differential protein expression of BOP1 and EXOSC4 in HCC tissues, the relatively small sample size (48 HCC cases with paired adjacent tissues) may limit the generalizability of these findings. Moreover, the absence of functional experiments prevents us from establishing their causal roles in HCC progression or immune regulation. Further studies involving larger independent cohorts and functional validation are warranted. Third, computational predictions based on TIDE, TMB, and immune infiltration analyses cannot substitute for actual clinical immunotherapy outcomes. The lack of an independent cohort of HCC patients treated with immune checkpoint inhibitors limits the validation of the clinical relevance and potential predictive value of these findings. Future studies involving ICI-treated patients are warranted to validate these predictions and clarify their clinical implications.

## 5. Conclusions

The study associates RiboSis-related expression with distinct HCC cell states, molecular subtypes and inferred immune features. BOP1 and EXOSC4 showed higher tumor staining, and EXOSC4 varied by HBV status in the analyzed tissue microarray. Independent cohort validation, functional experiments and immunotherapy outcome data are required to establish clinical utility or mechanism.

## Figures and Tables

**Table 1:** Association between EXOSC4 immunohistochemical H-scores and clinicopathological characteristics in 48 patients with HCC.

Characteristics	Total (*n* = 48)	DAB H-Score *p* Value
Gender		0.541
Female	8	
Male	40	
Age		0.106
>60	16	
≤60	32	
Tumor size		0.733
>5 cm	35	
≤5 cm	13	
AFP		0.602
>20	20	
≤20	28	
HBV infection status		<0.001
Yes	36	
No	12	
Ki-67 (%)		0.128
<10	13	
10–30	30	
>30	5	
TNM.stage		0.146
I	15	
II	10	
III	20	
IV	3	
MVI.stage		0.37
M0	27	
M1	18	
M2	3	

**Table 2:** Association between BOP1 immunohistochemical H-scores and clinicopathological characteristics in 48 patients with HCC.

Characteristics	Total (*n* = 48)	DAB H-Score *p* Value
Gender		0.471
Female	8	
Male	40	
Age		0.662
>60	16	
≤60	32	
Tumor size		0.451
>5 cm	35	
≤5 cm	13	
AFP		0.754
>20	20	
≤20	28	
HBV infection status		0.651
Yes	36	
No	12	
Ki-67 (%)		0.991
<10	13	
10–30	30	
>30	5	
TNM.stage		0.411
I	15	
II	10	
III	20	
IV	3	
MVI.stage		0.508
M0	27	
M1	18	
M2	3	

## Data Availability

The dataset(s) supporting the conclusions of this article is(are) included within the article (and its Supplementary Materials).

## Supplementary Materials

The following supporting information can be downloaded at: https://www.mdpi.com/article/10.3390/biology15212034/s1, Table S1: RiboSis-related genes. Table S2: Single-cell clustering and annotation results. Table S3: DEGs between RiboSis-related hepatocyte subclusters. Table S4: Enrichment of DEGs along pseudotime trajectory branches. Table S5: DEGs between GLUL^+^ hepatocytes and other subclusters. Table S6: DEGs between HCC and normal samples (transcriptome). Table S7: RiboSis-related genes in HCC. Table S8: GO enrichment of hub genes. Table S9: KEGG pathway enrichment of hub genes. Table S10: Clinical characteristics of the two RiboSis-associated subtypes in TCGA-LIHC. Table S11: DEGs between two HCC subtypes. Table S12: Most significantly altered pathways between subtypes. Table S13: Differential pathway enrichment between RiboSis subtypes. Table S14: Infiltration levels of 28 immune cell types in HCC. Table S15: Drug sensitivity across RiboSis subtypes. Table S16: TIDE scores across subtypes. Figure S1: Gene selection and molecular subtyping. Figure S2: Selection of Hub Genes Using Machine Learning Approaches. Figure S3: Stratification of HCC samples based on RiboSis–related genes. Figure S4: Analysis of immune cell infiltration in HCC samples. Figure S5: Drug sensitivity and TMB analysis. Figure S6: Analysis of immune checkpoint–related features across RiboSis subtypes. Figure S7: Univariate Cox associations between hub gene expression and overall survival across cancer types.
