# Supplementary material for: Integrative Single-Cell and Bulk Transcriptomic Analysis Reveals Ribosome Biogenesis-Associated Molecular Subtypes and Immune Characteristics in Hepatocellular Carcinoma
Source: Biology (Basel). 2026 Nov 8;15(21):2034. doi: 10.3390/biology15212034 (PMC13762202; doi:10.3390/biology15212034)

Supplementary figures

Figure S1: Gene selection and molecular subtyping.

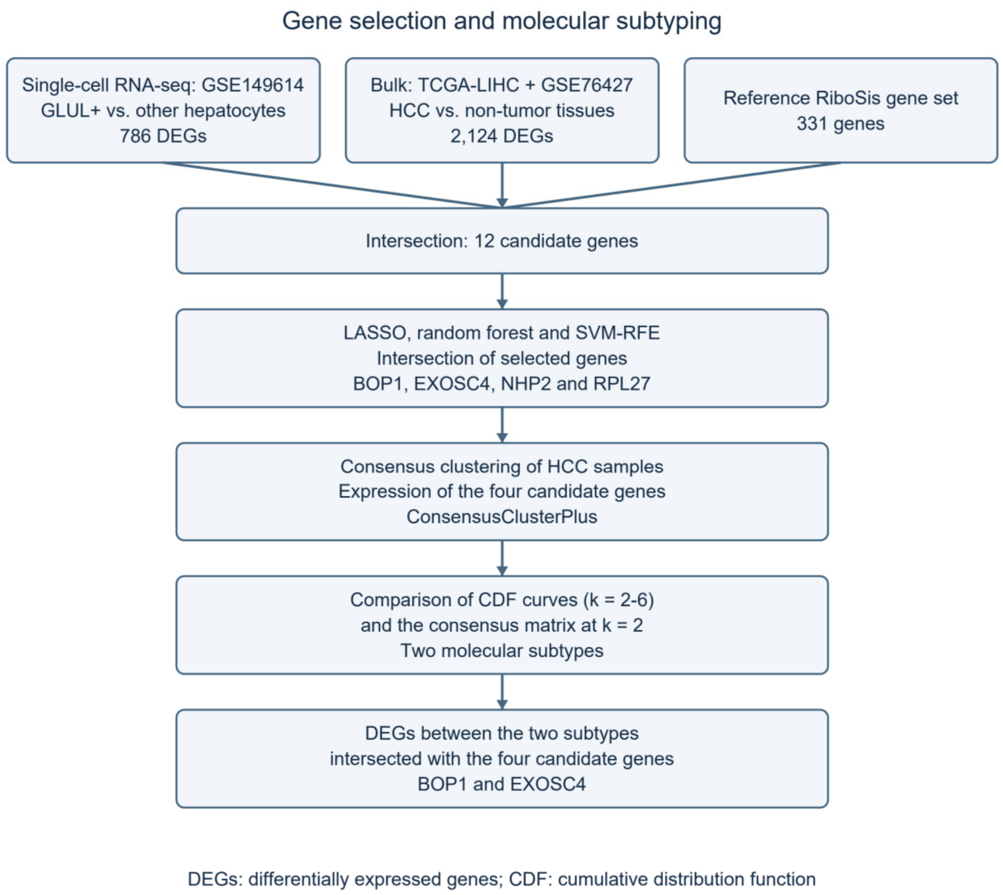

Figure S2: Selection of Hub Genes Using Machine Learning Approaches. **(A)** LASSO coefficient profiles of the candidate genes. The X-axis represents the log(lambda) sequence, and the Y-axis represents the coefficient values. **(B)** Cross-validation error versus lambda in the LASSO regression. Each error bar represents the confidence interval for a given lambda. **(C)** Error rate of the random forest model as a function of the number of classification trees. **(D)** Top 10 feature genes ranked by mean decrease accuracy and mean decrease Gini in the random forest algorithm. **(E)** Accuracy curve of the SVM-RFE algorithm during feature selection. **(F)** Error rate curve of the SVM-RFE algorithm during feature selection. **(G)** Venn diagram illustrating the final hub genes identified by the intersection of three machine learning methods. Source data were obtained from TCGA-LIHC and GEO dataset GSE76427.

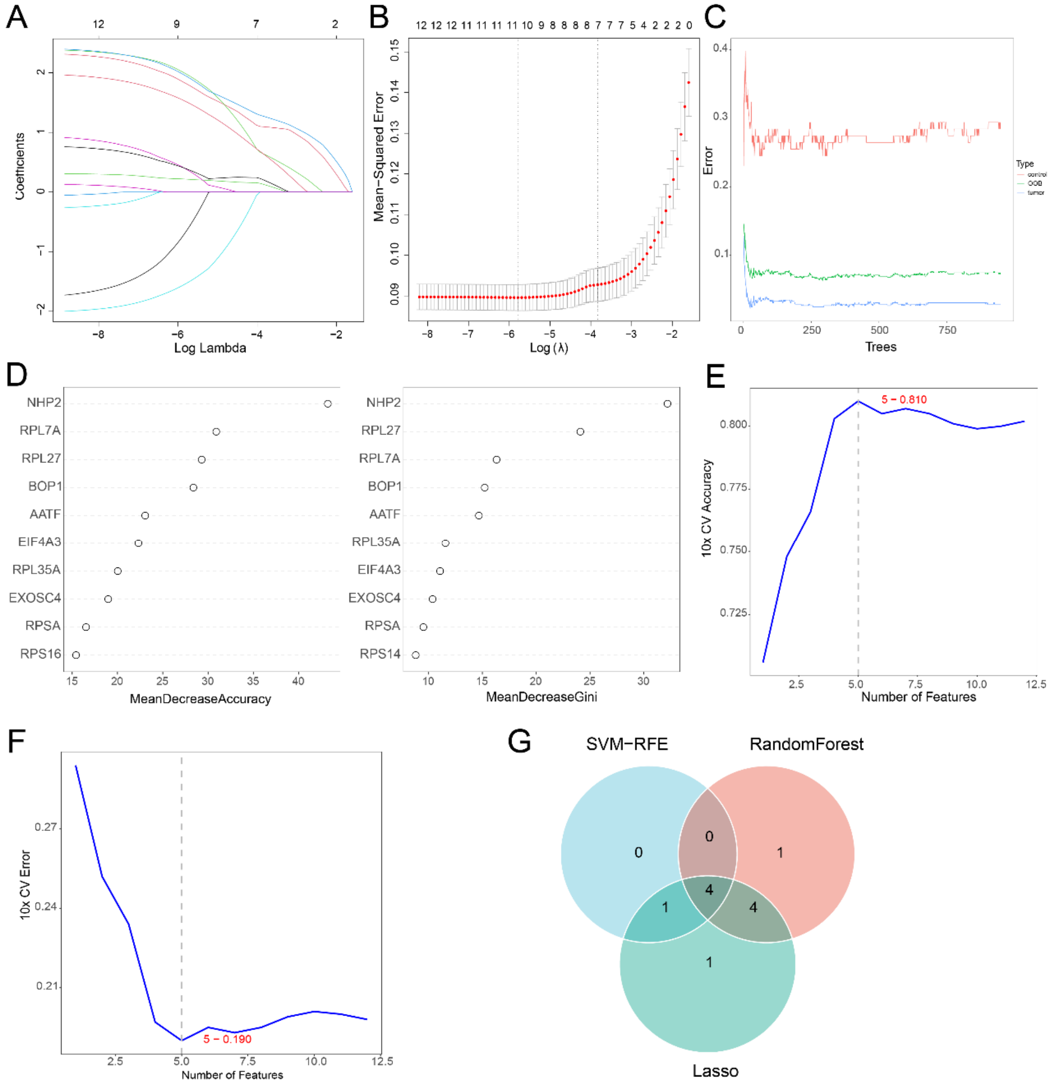

Figure S3: Stratification of HCC samples based on RiboSis–related genes. **(A)** Cumulative distribution function (CDF) curve from consensus clustering. **(B)** HCC sample classification derived from RiboSis–related gene expression. **(C)** Expression patterns of hub genes across different subtypes. **(D)** ROC curves of hub genes for subtype discrimination. Source data were obtained from TCGA-LIHC and GEO dataset GSE76427.

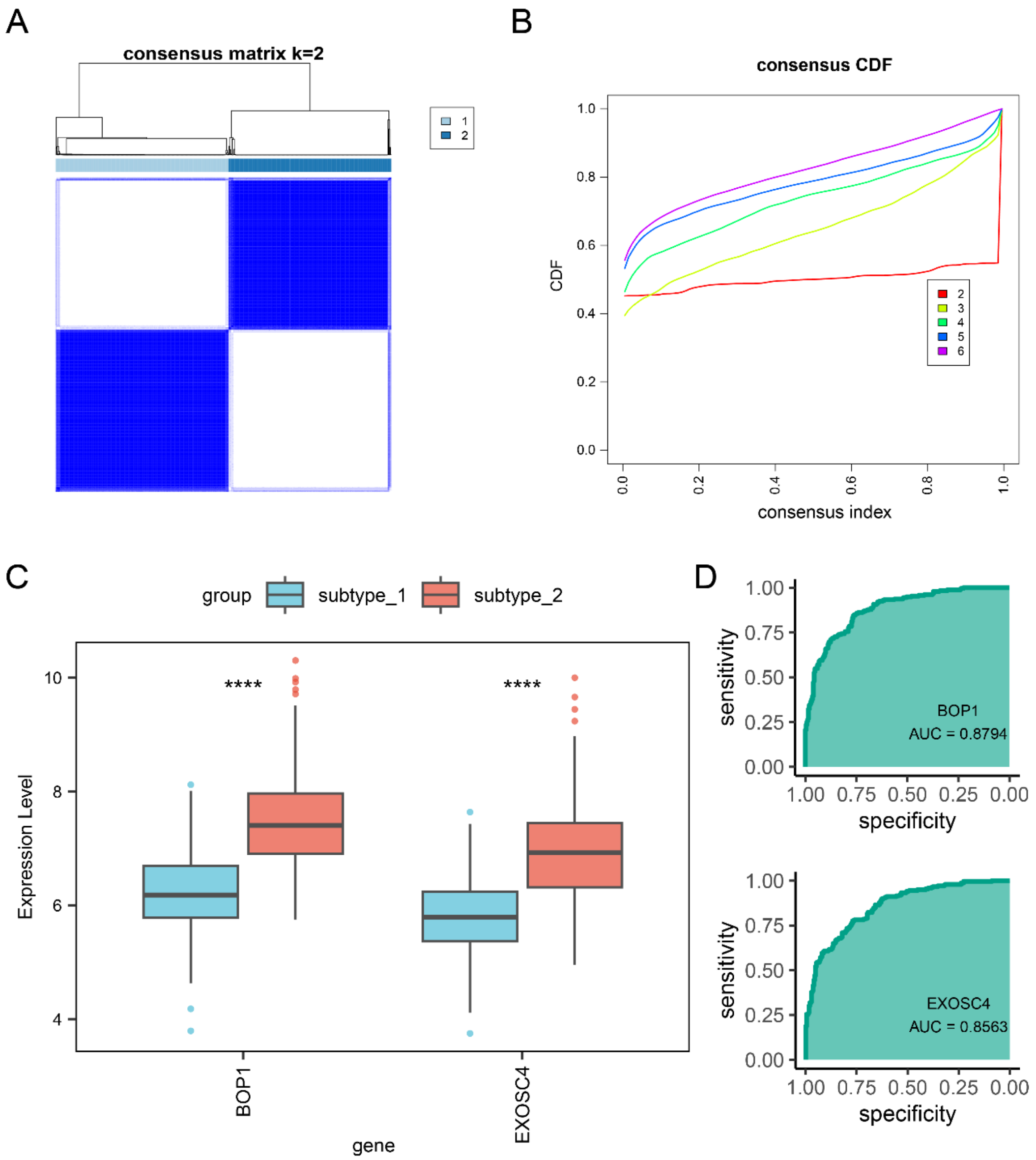

Figure S4: Analysis of immune cell infiltration in HCC samples. **(A)** Infiltration levels of 28 immune cell types across HCC samples. **(B)** Correlation heatmap of infiltration levels among immune cell types. Source data were obtained from TCGA-LIHC and GEO dataset GSE76427.

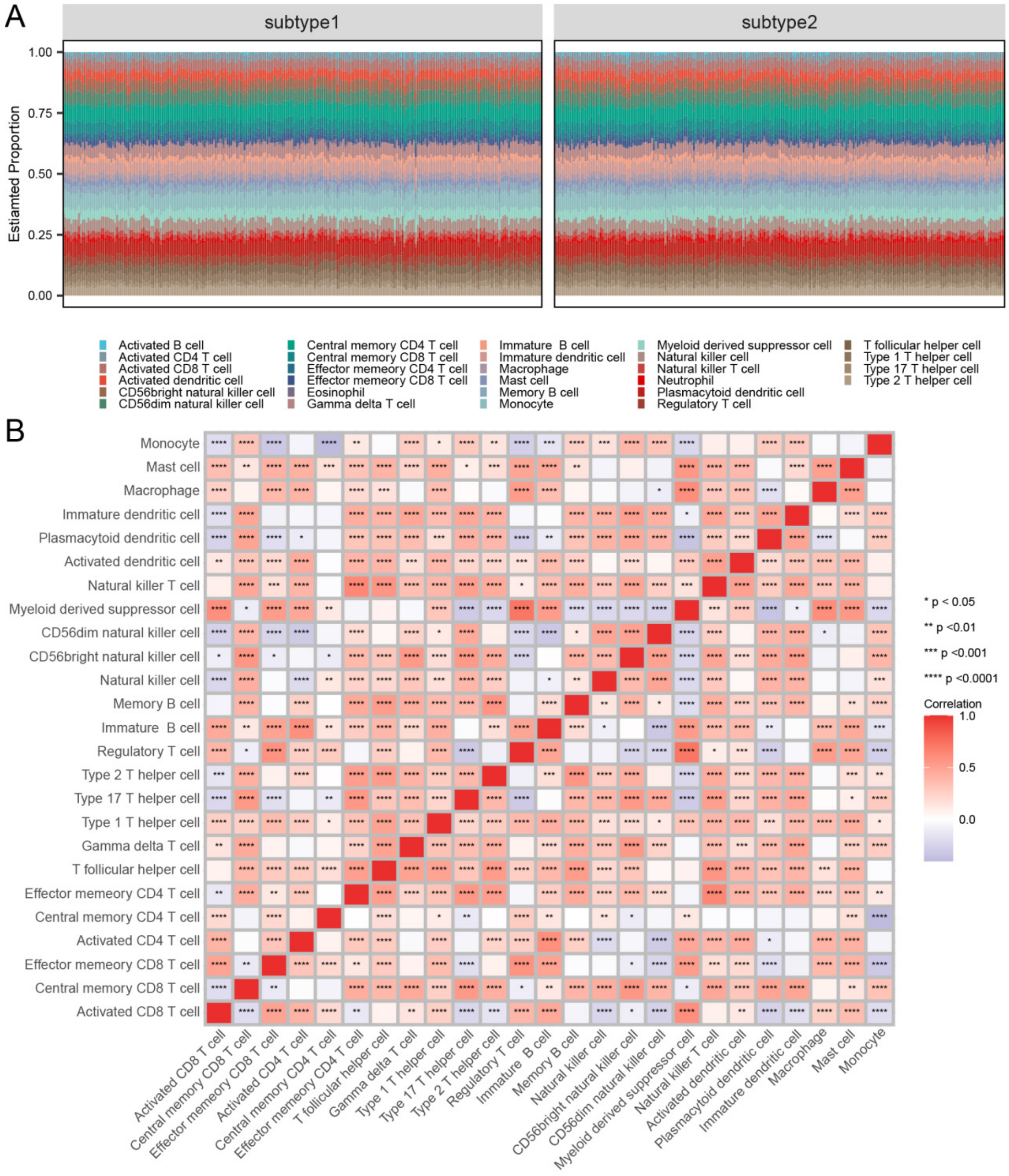

Figure S5. Drug sensitivity and TMB analysis. **(A)** Top 20 most frequently mutated genes in different RiboSis–related subtypes. **(B)** Difference in TMB between subtypes. Subtype2 shows a significantly higher mutation frequency than Subtype1 ($p < 0.05$). **(C–K)** Violin plots showing differential sensitivity of subtypes to specific compounds: (C) AZD7762_1022(CHK1/CHK2 inhibitor), (D) AZD8055_1059(mTORC1/2 inhibitor), (E) Dactolisib_1057, (F) BI−2536_1086(PLK1 inhibitor), (G) BMS−754807_2171(IGF-1R/IR inhibitor), (H) Daporinad_1248, (I) Rapamycin_1084, (J) Sepantronium bromide_1941, (K) Vinorelbine_2048. Mutation data were obtained from TCGA-LIHC, and drug-sensitivity predictions were based on GDSC data.

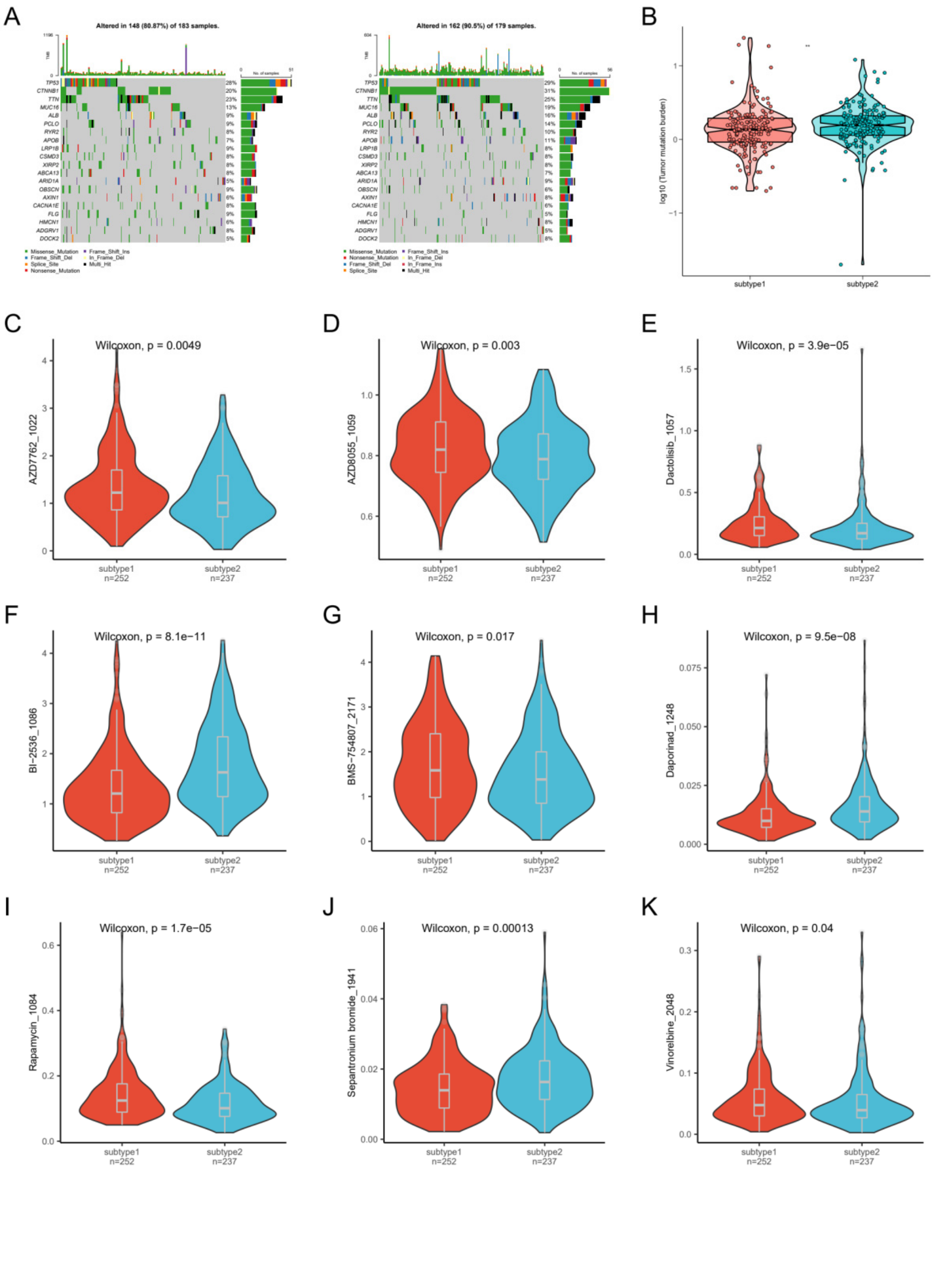

Figure S6: Analysis of immune checkpoint–related features across RiboSis subtypes**.** **(A)** TIDE, Exclusion, and Dysfunction scores among the subtypes. **(B)** Immunotherapy response in each subtype. **(C)** Expression profiles of immune checkpoint molecules across subtypes. Source data were obtained from TCGA-LIHC and GEO dataset GSE76427; immunotherapy response was predicted using TIDE.

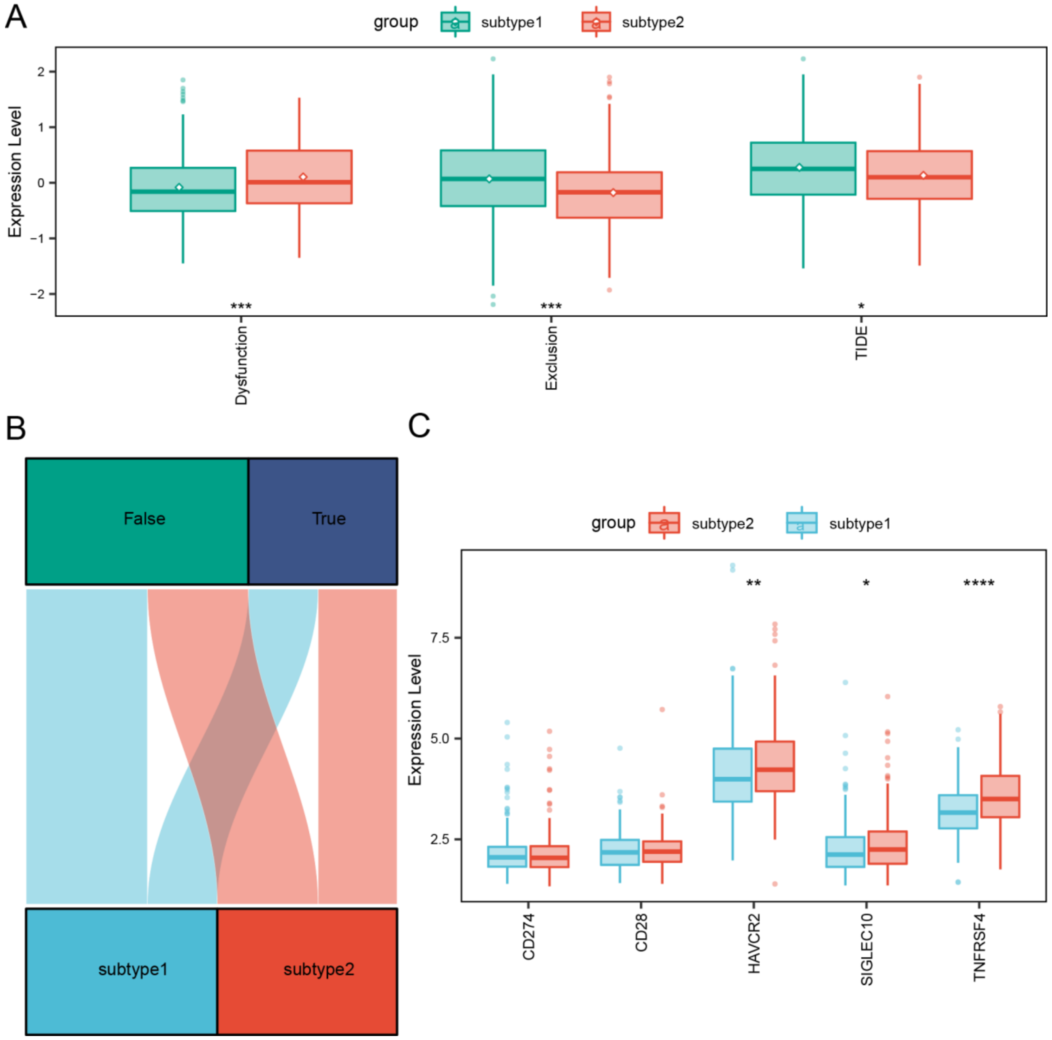

Figure S7: Univariate Cox associations between hub gene expression and overall survival across cancer types. **(A)** Forest plot of univariate Cox regression analysis for EXOSC4 across multiple cancer types. **(B)** Forest plot of univariate Cox regression analysis for BOP1 across multiple cancer types. Pan-cancer survival data were obtained from TCGA.

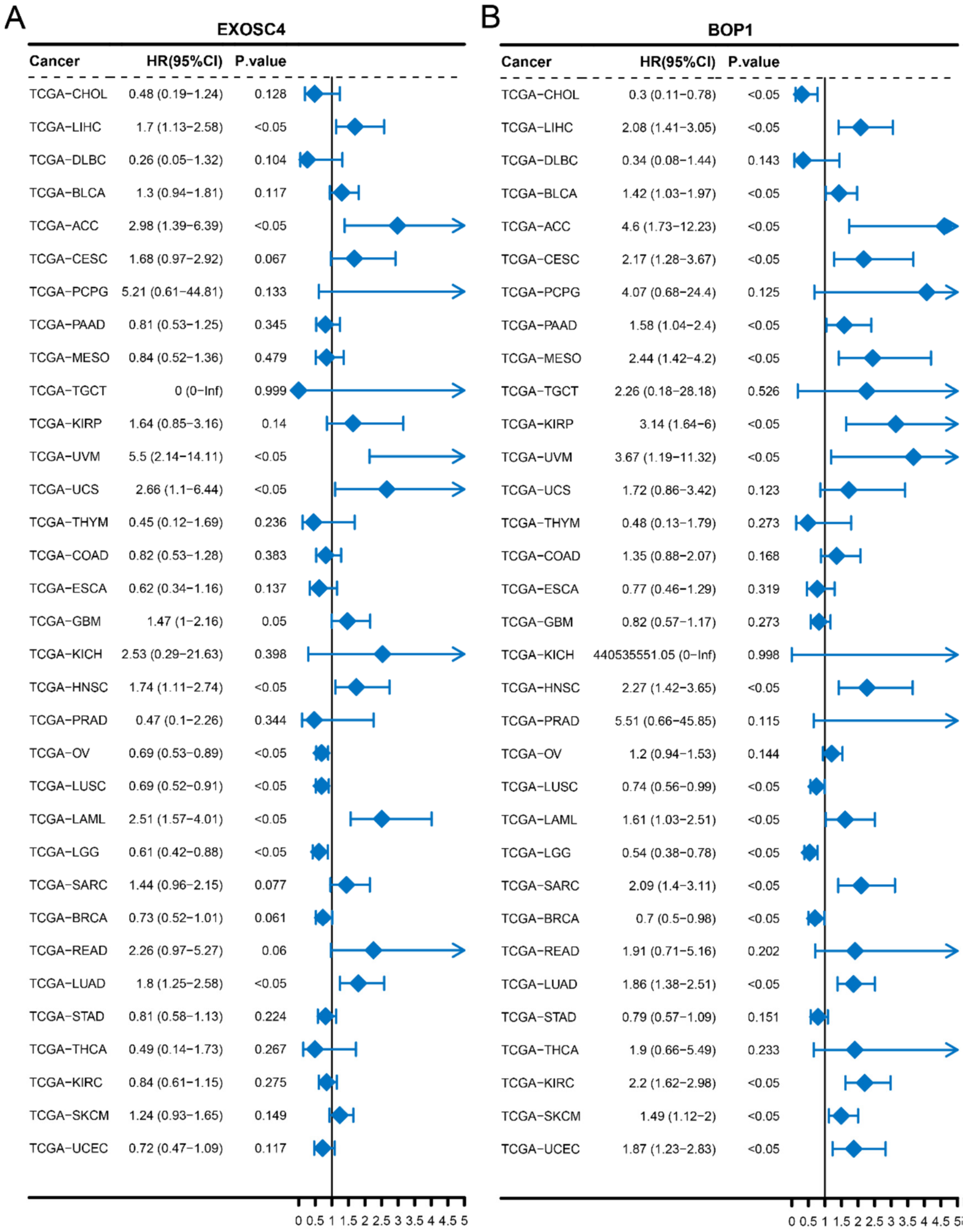

Supplement: Supplementary file 1 [file biology-15-02034-s001.zip › Supplementary figures.pdf]
