# Supplementary material for: Integrative Single-Cell and Bulk Transcriptomic Analysis Reveals Ribosome Biogenesis-Associated Molecular Subtypes and Immune Characteristics in Hepatocellular Carcinoma
Source: Biology (Basel). 2026 Nov 8;15(21):2034. doi: 10.3390/biology15212034 (PMC13762202; doi:10.3390/biology15212034)
Supplement: Supplementary file 1 [file biology-15-02034-s001.zip › Supplementary tables/Table S1 RiboSis -related genes..pdf]

RiboSis gene
METTL15P1
RCL1
MPHOSPH10
MPHOSPH6
POP7
NPM3
NSA2
EMG1
C1D
MYBBP1A
DDX17
NOP56
RPP30
RPP38
TBL3
POP4
RPP40
UTP14A
WDR3
EBNA1BP2
DDX52
KRR1
PWP1
LSM6
RPL35
ERI2
RPUSD1
EXOSC8
HELQ
BUD23
MALSU1
TSR3
FTSJ3
EXOSC6
PIH1D2
MRPL10
MTERF4
NUDT16
WDR36
RPL10L
SRFBP1
BMT2
NSUN5P1

NOP9
DDX3X
DDX10
DKC1
METTL15
ERCC2
FBL
LTO1
RPLP0P6
XRN2
FASTKD2
DIS3
DHX30
PDCD11
EXOSC7
RRP1B
WDR43
MDN1
RRS1
RRP12
BOP1
RRP8
EXOSC2
PES1
MTREX
GTPBP4
FRG1
DCAF13
NOL11
NGDN
NSUN5P2
ZNF658
NOC2L
RSL1D1
MTG2
SENP3
ERAL1
AATF
VCX
UTP25
PELP1
RPUSD2
DIMT1
UTP20

RRP7A
GLUL
RPUSD3
RPL7L1
NOB1
ZCCHC4
METTL5
MRPL22
DROSHA
GTF3A
ABT1
GNL2
MRM2
NOP53
DDX51
FBLL1
ISG20
EIF6
NSUN4
RPSA
RBIS
GTF2H5
RPL10A
SNU13
NOP2
NPM1
NUP88
NVL
PA2G4
GEMIN4
MTERF3
EXOSC3
EXOSC1
RRP15
RPS27L
NMD3
FCF1
MRPS7
RRNAD1
UTP18
TFB1M
MRPS2
UTP11
SBDS

RPL26L1
MRTO4
RSL24D1
DDX47
POP5
NIP7
NOP16
TRMT112
SIRT7
ESF1
NOP58
WBP11
PIN4
EXOSC9
EXOSC10
YBEY
GAR1
NLE1
DHX29
EXOSC4
GNL3L
DDX49
DDX56
WDR74
ZNHIT6
WDR55
TEX10
RPP25
PAK1IP1
TRMT61B
PIH1D1
HEATR3
NOL8
MRPL20
HEATR1
SDAD1
SHQ1
MRM3
NAT10
IMP3
BRIX1
LSG1
NOP10
THUMPD1

CHD7
LYAR
NHP2
DDX27
NSUN5
TSR1
WDR12
RIOK2
DDX28
UTP6
PRKDC
PPAN
EXOSC5
UTP3
REXO4
PTEN
WDR18
USP36
DHX37
PWP2
RAN
RMRP
RNASEL
ABCE1
RPL3
RPL3L
RPL5
RPL6
RPL7
RPL7A
RPL10
RPL11
RPL23A
RPL24
RPL26
RPL27
RPL35A
RPL38
RPLP0
RPS2
RPS5
RPS6
RPS7
RPS8

RPS9
RPS10
RPS14
RPS15
RPS16
RPS17
RPS19
RPS21
RPS24
RPS25
RPS27
RPS28
NSUN3
TFB2M
TENT4B
NOM1
METTL17
DDX31
MRPS11
MRPL36
MRPL1
NOL6
KRI1
SURF6
SUV39H1
BYSL
C1QBP
TSC1
XPO1
XRCC5
ERI3
DDX54
NOC4L
METTL16
EFL1
NOL9
RBFA
MRM1
NOL10
RPF1
ISG20L2
LAS1L
RIOK1
EIF2A

WDR75
UTP15
RPF2
TRAF7
UTP23
NIFK
CUL4B
CUL4A
MAK16
MPV17L2
RIOX2
RPUSD4
UTP4
LTV1
FAM207A
RRP1
GTPBP10
NOP14
RIOK3
KAT2B
RRP36
DDX18
TSR2
ZNF622
RPL14
ERI1
SART1
RRP9
RRP7BP
DDX21
FDXACB1
NOLC1
NAF1
WDR46
HELB
IMP4
RPS10P5
ZNHIT3
UTP14C
EIF4A3
BMS1
URB2
URB1
POLR1G

SPIN1
GTF3C6
DDX11
MTOR
ANG
CAVIN1
BRF1
GTF3C1
GTF3C2
MARS1
NCL
MACROH2A2
POLR1E
IPPK
SMARCA4
SMARCB1
TCOF1
TP53
POLR1B
TAF1B
GTF3C5
GTF3C4
GTF3C3
MACROH2A1
