# Supplementary material for: Integrative Single-Cell and Bulk Transcriptomic Analysis Reveals Ribosome Biogenesis-Associated Molecular Subtypes and Immune Characteristics in Hepatocellular Carcinoma
Source: Biology (Basel). 2026 Nov 8;15(21):2034. doi: 10.3390/biology15212034 (PMC13762202; doi:10.3390/biology15212034)
Supplement: Supplementary file 1 [file biology-15-02034-s001.zip › Supplementary tables/Table S2 Single-cell clustering and annotation results..pdf]

"celltype"	"marker"
"Hepatocyte"	"ALB"
"Hepatocyte"	"SERPINA1"
"Hepatocyte"	"HNF4A"
"Hepatocyte"	"EPCAM"
"T_NK"	"CD3D"
"T_NK"	"CD3E"
"T_NK"	"NKG7"
"Myeloid"	"CD68"
"Myeloid"	"CD14"
"Myeloid"	"CD163"
"Myeloid"	"CD1C"
"B_cell"	"IGHG1"
"B_cell"	"JCHAIN"
"B_cell"	"CD79A"
"Endothelial"	"VWF"
"Endothelial"	"PECAM1"
"Endothelial"	"FCGR2B"
"Fibroblast"	"ACTA2"
"Fibroblast"	"COL1A1"
"Fibroblast"	"COL1A2"
