# Supplementary material for: Integrative Single-Cell and Bulk Transcriptomic Analysis Reveals Ribosome Biogenesis-Associated Molecular Subtypes and Immune Characteristics in Hepatocellular Carcinoma
Source: Biology (Basel). 2026 Nov 8;15(21):2034. doi: 10.3390/biology15212034 (PMC13762202; doi:10.3390/biology15212034)
Supplement: Supplementary file 1 [file biology-15-02034-s001.zip › Supplementary tables/Table S7 RiboSis-related genes in HCC..pdf]

RPS14
RPL27
BOP1
NHP2
EXOSC4
RPL35A
RPL7A
AATF
EIF4A3
RPS16
RPS6
RPSA
